# Real-time imaging of glutamate clearance reveals normal striatal uptake in Huntington disease mouse models

**DOI:** 10.1038/ncomms11251

**Published:** 2016-04-07

**Authors:** Matthew P. Parsons, Matthieu P. Vanni, Cameron L. Woodard, Rujun Kang, Timothy H. Murphy, Lynn A. Raymond

**Affiliations:** 1 Brain Research Centre and Djavad Mowafaghian Centre for Brain Health, Department of Psychiatry, University of British Columbia, 2255 Wesbrook Mall, Vancouver, British Columbia, Canada V6T 1Z3

## Abstract

It has become well accepted that Huntington disease (HD) is associated with impaired glutamate uptake, resulting in a prolonged time-course of extracellular glutamate that contributes to excitotoxicity. However, the data supporting this view come largely from work in synaptosomes, which may overrepresent nerve-terminal uptake over astrocytic uptake. Here, we quantify real-time glutamate dynamics in HD mouse models by high-speed imaging of an intensity-based glutamate-sensing fluorescent reporter (iGluSnFR) and electrophysiological recordings of synaptically activated transporter currents in astrocytes. These techniques reveal a disconnect between the results obtained in synaptosomes and those *in situ*. Exogenous glutamate uptake is impaired in synaptosomes, whereas real-time measures of glutamate clearance in the HD striatum are normal or even accelerated, particularly in the aggressive *R6/2* model. Our results highlight the importance of quantifying glutamate dynamics under endogenous release conditions, and suggest that the widely cited uptake impairment in HD does not contribute to pathogenesis.

## Abstract (web summary)

Huntington disease (HD) has been linked via biochemical uptake assays to impaired glutamate clearance and resultant excitotoxicity. Here, utilizing a fluorescent reporter, the authors measure real-time glutamate dynamics in mouse model HD brain slices and find normal or even accelerated glutamate clearance.

## Graphical abstract

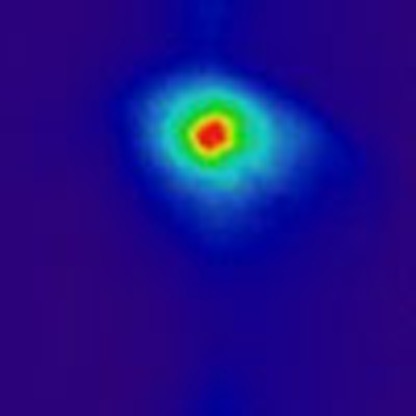

Glutamate transporters are responsible for the rapid uptake of extracellular glutamate following synaptic release. This ensures a low basal level of extracellular glutamate necessary to achieve a high signal-to-noise ratio during neurotransmission, and prevents overactivation of neuronal glutamate receptors that can promote cell-death signalling^1^. Various lines of evidence, notably from biochemical uptake assays in synaptosomal preparations, have suggested that several neurological conditions are characterized by impaired transporter-mediated glutamate uptake. This reduced capacity of synaptosomes to take up exogenous glutamate has been extrapolated to indicate a prolonged temporal profile of extracellular glutamate following synaptic release, thereby enhancing neuronal susceptibility to excitotoxic cell death^2^,^3^,^4^,^5^,^6^,^7^,^8^. As a result, the enhancement of transporter-mediated uptake is understood to be a viable therapeutic approach for a number of conditions, particularly Huntington disease (HD).

HD is a neurodegenerative disorder caused by a CAG repeat expansion in the gene encoding the huntingtin protein^9^. This mutation gives rise to a clinical triad of motor, cognitive and psychiatric symptoms as well as progressive brain atrophy that is particularly striking in the striatum. Huntingtin interacts with hundreds of proteins^10^, and the mutant protein has been implicated in altered protein and organelle trafficking, changes in cellular metabolism, disrupted mitochondrial function and calcium homeostasis, transcriptional dysregulation and synaptic dysfunction^11^. In addition, the earliest animal models of HD relied on intrastriatal injections of glutamate receptor agonists^12^,^13^, and evidence indicates that striatal neurons show increased susceptibility to glutamate-mediated excitotoxicity in early HD^14^. Several studies demonstrate a reduced uptake capacity when HD striatal tissue is exposed to exogenous glutamate or aspartate on a timescale of minutes (see Table 1 for references). These data have promulgated the view that glutamate uptake, particularly astrocytic uptake mediated by glutamate transporter-1 (GLT-1), is impaired in HD, resulting in extracellular glutamate build-up and excitotoxic signalling^15^,^16^,^17^,^18^,^19^,^20^. However, emerging data convincingly demonstrate that the uptake of externally supplied substrate in the brains slices and synaptosomal preparations largely occurs in the nerve terminals rather than in astrocytes^21^,^22^. This is an important finding, as a much higher density of uptake sites is found on astrocytes than on neurons^1^,^22^ and, accordingly, there appears to be a much greater physiological role of astrocytic uptake in comparison with nerve terminal uptake^21^. Together, these data highlight the need to revisit the well-accepted view of an uptake impairment in HD, as no study to date has tested whether the HD mutation influences the time course of extracellular glutamate following synaptic release.

Here, we use a rapid, intensity-based glutamate-sensing fluorescent reporter (iGluSnFR)^23^ to visualize the spatiotemporal dynamics of extracellular glutamate in the HD striatum following evoked synaptic release. By virally expressing iGluSnFR under the control of a neuronal promoter, we demonstrate that striatal neurons in HD are not exposed to a prolonged time course of extracellular glutamate following synaptic release, despite a significant reduction in ^3^H-labelled glutamate uptake in HD striatal synaptosomes as quantified by a standard biochemical assay. Surprisingly, endogenous glutamate clearance in the brain slices, as measured both by high-speed iGluSnFR imaging and electrophysiological measures of transporter-mediated currents recorded from striatal astrocytes, is consistently accelerated in the well-characterized aggressive *R6/2* mouse model of HD. Our data suggest that biochemical measurements of exogenous glutamate uptake capacity do not necessarily correlate with glutamate clearance dynamics *in situ*, and highlight the need to re-evaluate our views on the contribution of, and therapeutic potential of targeting, glutamate transporters in disease.

## Results

### Using iGluSnFR to quantify endogenous glutamate clearance

**Figure 1:**
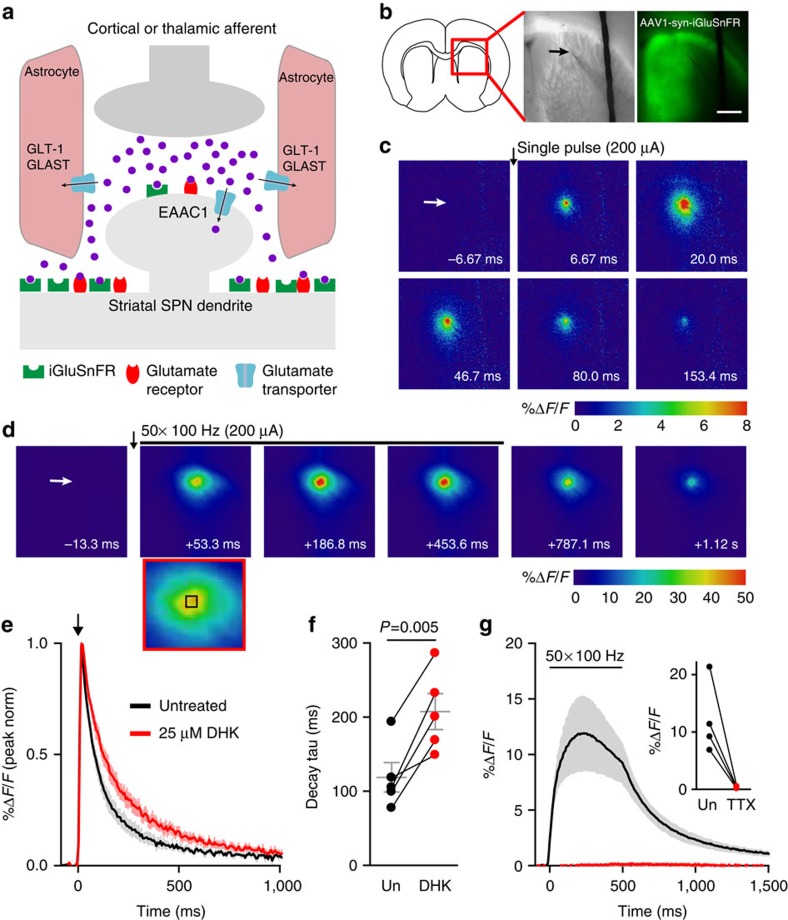
Characterization of iGluSnFR responses in the dorsal striatum evoked by electrical stimulation. (**a**) Simplified schematic depicting the basic spatial organization of a glutamatergic synapse in the striatum. For the majority of the experiments in the present study, iGluSnFR was expressed exclusively on neurons to measure the relative time course of extracellular glutamate sensed by these neurons. Glutamate uptake can occur through glutamate transporter-1 (GLT-1) and glutamate-aspartate transporter (GLAST), located predominantly on astrocytes, or through excitatory amino acid carrier 1 (EAAC1), located predominantly on neurons^34^. (**b**) Typical experimental setup showing the extent of iGluSnFR expression in the striatum (shown in green on the right) and the approximate placement of the stimulating electrode to evoke local glutamate release (arrow). Expression was generally strong along entire dorsal striatum immediately ventral to the corpus callosum. (**c**,**d**) Representative heat-maps showing specific frames of representative iGluSnFR responses following a single stimulus at 200 μA (**c**) or 50 pulses at 100 Hz (200 μA; **d**). The red-bordered frame in **d** is a magnified view of the panel immediately above it, with a 4 × 4 black box drawn to depict the region of interest corresponding to the region of maximal iGluSnFR signal that was used for quantification. (**e**) Grouped data (±s.e.m.) demonstrating the effect of dihydrokainic acid (DHK; 25 μM for 10 min) on iGluSnFR decay tau. Each response was normalized (norm) to its peak to emphasize the slowing effect DHK has on iGluSnFR decay. DHK had no significant effect on response size (before DHK: 4.6±1.7%ΔF/F; after DHK: 4.4±1.9%ΔF/F, *n*=5, *P*=0.81, paired *t*-test). (**f**) Paired values for the experiments in **e**. In all the cases, 25 μM DHK resulted in a robust increase in iGluSnFR decay time (paired *t*-test). (**g**) Grouped data showing that iGluSnFR responses, even following high-frequency stimulation (50 pulses, 100 Hz, 200 μA), were completely blocked by tetrodotoxin (TTX). TTX is shown in red, untreated (Un) is shown in black. Scale bar, 1 mm in **b** and applies to all square panels in **b**,**c** and **d**.

Slow glutamate clearance as a result of dysfunctional transporter-mediated uptake has been associated with neuronal cell death in disease via pathogenic activation of ionotropic glutamate receptors located on neurons. For this reason, we were interested in quantifying the temporal dynamics of glutamate sensed at the surface of striatal neurons (Fig. 1a) in mouse models of HD. iGluSnFR was expressed under the control of the synapsin promoter by viral delivery (AAV2/1-synapsin-iGluSnFR) into the dorsal striatum. Glutamate release was evoked in acute brain slices by local placement of a tungsten stimulating electrode in the dorsal striatum (Fig. 1b), and widefield iGluSnFR responses were imaged at 150 Hz (6.67 ms frame-rate) with a CCD camera. Rapid iGluSnFR responses, quantified within a 4 × 4 pixel region localized to the maximal response site, were readily observed under a variety of stimulation conditions, including brief bursts of ≥2 pulses at 100 μA or a single pulse at ≥200 μA (Fig. 1c). A typical response to a longer high-frequency stimulation (HFS) protocol consisting of 50 pulses at 100 Hz is shown in Fig. 1d. Single pulses at 100 μA were generally too weak to reliably elicit a detectable response.

To assess the sensitivity of iGluSnFR to an impairment in transporter-mediated uptake, we bath applied a low concentration (25 μM; 10 min) of the GLT-1-specific inhibitor dihydrokainic acid (DHK). This resulted in a significant increase in the decay tau of evoked iGluSnFR responses (Fig. 1e,f), demonstrating the ability to detect relative changes in the temporal profile of extracellular glutamate with this experimental approach. All evoked responses were absent in the presence of TTX (1 μM; Fig. 1g, *n*=4), even when the slices were challenged with a HFS protocol (50 pulses, 100 Hz, 200 μA), indicating the action potential-dependent nature of the evoked signals. Despite resulting in larger peaks, increasing the number of pulses or the pulse intensity did not enhance the proportional spread of iGluSnFR signals from the central point of maximal release (Supplementary Fig. 2a–f), suggesting that the iGluSnFR response radius is unlikely to represent glutamate diffusion originating from the area of maximal release. Furthermore, the rise and decay kinetics of iGluSnFR responses were found to be nearly identical regardless of whether the 4 × 4 region of interest (ROI) was placed in the region of maximal glutamate release or 500 μm away from this central release site (Supplementary Fig. 2g,h), suggesting that electrical stimulation evokes a wide radius of simultaneous glutamate release in this preparation, at least as detected by the 6.67 ms frame-rate used here. Thus, all the future analyses were restricted to the central region of release adjacent to the stimulating electrode to optimize the signal-to-noise ratio.

### *In situ* glutamate clearance in *YAC128* mice

Numerous groups have demonstrated a reduction in GLT-1 mRNA and/or protein expression that associates with a significant reduction in glutamate uptake capacity in striatal tissue from HD mouse models (Table 1). For example, biochemical quantification of exogenous ^3^H-glutamate uptake in striatal synaptosomes from *YAC128* mice expressing full-length human mutant huntingtin demonstrates a significant deficit in GLT-1-mediated uptake at 3 months of age^17^, and we report here that this effect is also robust when measured in striatal synaptosomes isolated from 1-month-old *YAC128* mice (*FVB/N*: 35.1±4.3 nmol per mg per min, *YAC128*: 16.3±5.1 nmol per mg per min, *n*=6, *P*<0.0001, paired *t*-test; Table 1). To determine whether these biochemical findings translate to a prolonged time course of extracellular glutamate following endogenous release, we examined evoked iGluSnFR responses in the striatum from wild-type (WT; *FVB/N*) and age-matched *YAC128* mice, before the onset of an overt motor phenotype (Supplementary Table 1). For these experiments, iGluSnFR was expressed under the control of the synapsin promoter to quantify relative differences in glutamate dynamics sensed at the extracellular surface of striatal neurons. iGluSnFR surface expression was not affected by the presence of mutant huntingtin (Supplementary Fig. 3a–c), and acute brain slices from WT and *YAC128* mice responded similarly to known concentrations of exogenous glutamate (Supplementary Fig. 3d), demonstrating that sensor expression, membrane insertion and response to glutamate was not different between genotypes.

**Figure 2:**
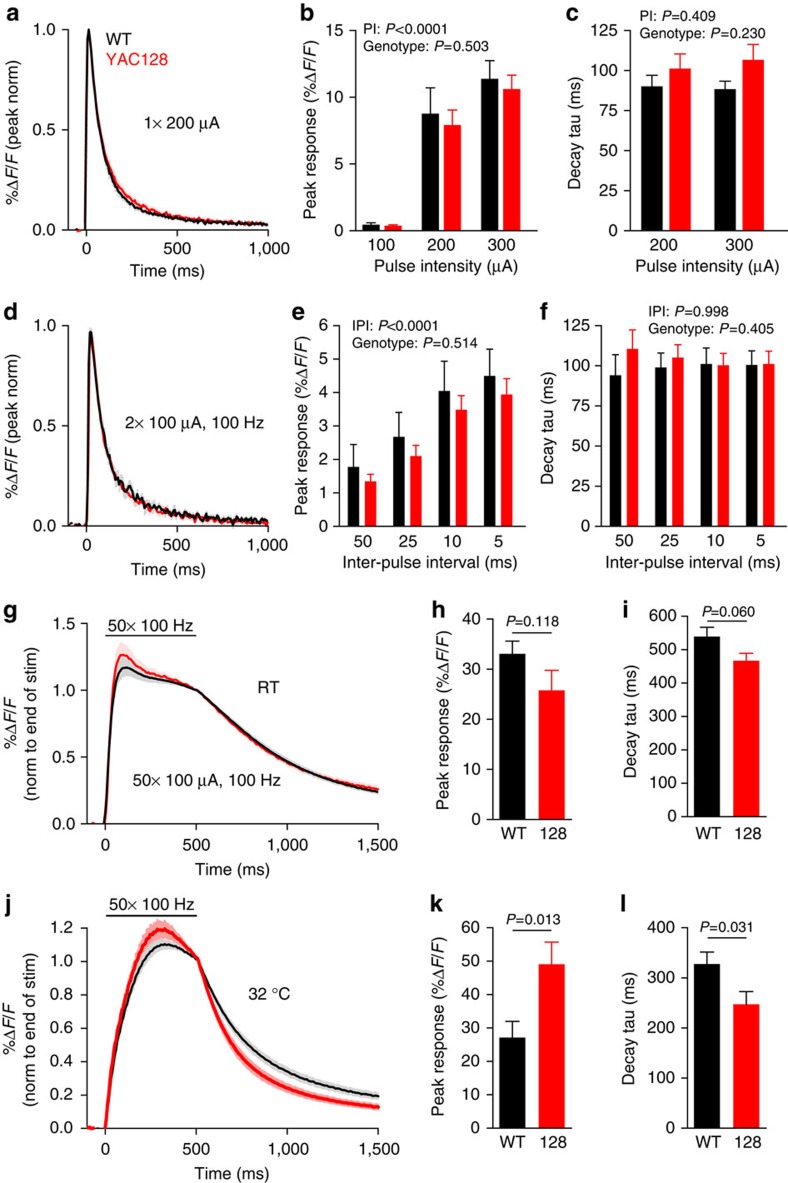
Glutamate clearance *in situ* is not impaired in *YAC128* dorsal striatum at 2 months of age. (**a**) Grouped data showing the average iGluSnFR response (±s.e.m.) to a single pulse (200 μA) in the dorsal striatum from both WT (*FVB/N*) and age-matched *YAC128* mice. The responses are normalized (norm) to their peaks to simplify comparisons of decay kinetics. (**b**,**c**) Bar graphs showing iGluSnFR response amplitude (**b**; WT *n*=12, 9, 9 from left to right; *YAC128 n*=14, 12, 12 from left to right, two-way ANOVA) and decay time (**c**; WT *n*=9 per bar; *YAC128 n*=12 per bar, RM two-way ANOVA) evoked by single pulses at increasing intensities. Response size but not decay is dependent on the pulse intensity (PI). No genotype differences are seen in either peak or decay. The responses were barely detectable following a single pulse at 100 μA and therefore no decay tau was calculated for this condition. (**d**) Average-normalized iGluSnFR response to a paired-pulse stimulation in the dorsal striatum from both WT (*FVB/N*) and age-matched *YAC128* mice. (**e**,**f**) Bar graphs showing iGluSnFR response amplitude (**e**) and decay time (**f**) in response to paired pulses at decreasing inter-pulse intervals (IPI; WT *n*=12 per bar; *YAC128 n*=14 per bar). The response size but not decay is dependent on IPI (RM two-way ANOVA). No genotype differences are seen in either peak or decay (RM two-way ANOVA). (**g**) Average iGluSnFR response at room temperature (RT) to a high-frequency stimulation (HFS) consisting of 50 pulses over 500 ms. The responses are normalized to their value at the end of stimulation to simplify comparisons of the decay time course. (**h**,**i**) Bar graphs demonstrating no significant differences between WT (*n*=21) and *YAC128* (*n*=17) for iGluSnFR amplitude (**h**) and decay (**i**) following HFS at RT. (**j**–**l**) Same as **g**–**i**, except that experiments were performed at 32 °C. Under these conditions, iGluSnFR responses from *YAC128* striatum (*n*=11) were significantly larger (**k**) and decayed faster (**l**) in comparison with WT (*n*=13). An unpaired *t*-test was used for **h**,**i**,**k** and **l**.

Contrary to our hypothesis, iGluSnFR response profiles were strikingly similar between genotypes when glutamate release was evoked by a variety of stimulation conditions at room temperature, including a HFS challenge (Fig. 2a–i). As both glutamate clearance and iGluSnFR dissociation kinetics are likely temperature dependent (Supplementary Fig. 4), we repeated the HFS experiment in slices perfused with ACSF clamped at a near-physiological temperature that also maintains optimal slice health (32±1 °C). Under such conditions, iGluSnFR fluorescence decayed faster in *YAC128* striatum (Fig. 2j,l) despite larger peak amplitudes (Fig. 2k). Thus, we are able to detect a robust glutamate uptake deficit in *YAC128* striatum through the use of a standard biochemical assay, yet we observe no evidence of slower extracellular glutamate clearance associated with neurotransmission.

As local stimulation within the striatum can evoke glutamate release from both cortical and thalamic terminals as well as promote the release of various local neuromodulators, we also imaged glutamate dynamics in the presence of extensive pharmacological blockade to rule out any potential effects of neuromodulator release. The blockade of dopaminergic, cholinergic, GABAergic and metabotropic glutamatergic signalling, combined with competitive antagonism of AMPA and NMDA ionotropic glutamate receptors, had no effect on iGluSnFR decay curves (0.3±5.29% change in decay tau (τ) from the pre-treatment value, *n*=4 slices from two mice, *P*=0.93, paired *t*-test). This suggests that local stimulation does not interfere with measurements of iGluSnFR decay as a result of neuromodulator release. As competitive antagonism of AMPA and NMDA receptors did not accelerate iGluSnFR decay, it is also unlikely that glutamate buffering by ionotropic glutamate receptors interferes with measures of iGluSnFR decay.

**Figure 3:**
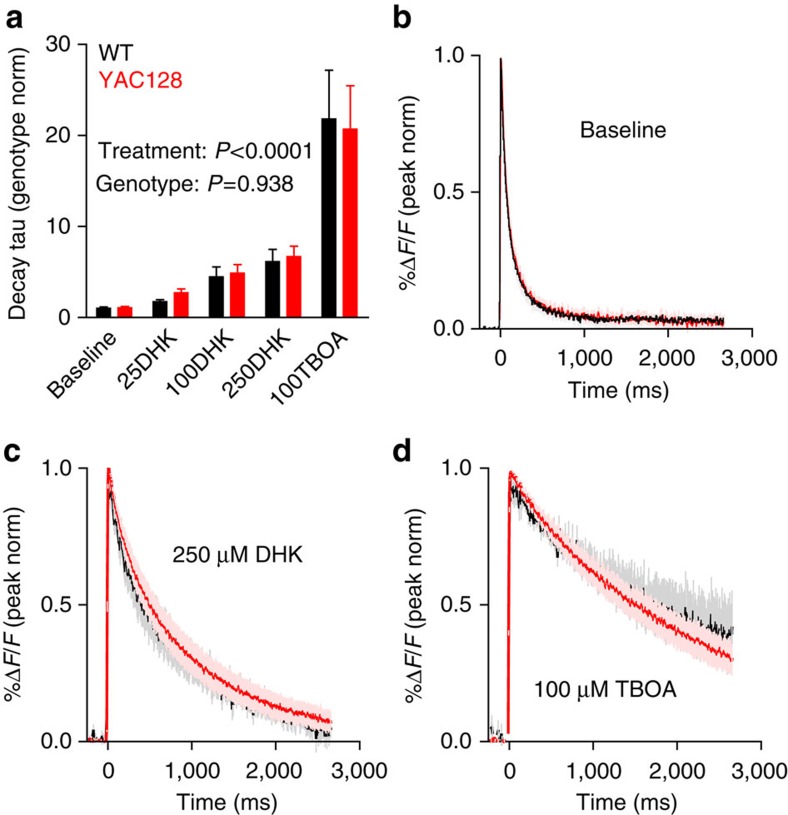
Pharmacological inhibition of GLT-1 reveals no impairment of GLT-1-mediated glutamate clearance in 2-month-old *YAC128* striatum. (**a**) Bar graph showing the fold-increase in iGluSnFR decay tau following a 10-min bath application of the GLT-1-specific blocker dihydrokainic acid (DHK; 25, 100 and 250 μM) or the non-selective glutamate transporter inhibitor threo-β-benzyloxyaspartic acid (TBOA; 100 μM). Responses were evoked by a single pulse (200 μA) and are normalized to the genotype average in baseline (untreated) conditions. (**b**–**d**) Average responses (±s.e.m.) for WT (black) and *YAC128* (red) in baseline conditions (**b**) and after 250 μM DHK (**c**) and 100 μM TBOA (**d**). The responses are normalized (norm) to their peaks to simplify comparisons of decay kinetics. WT *n*=5; *YAC128 n*=5; RM two-way ANOVA.

Previous studies highlight GLT-1 as the defective transporter in HD (see Table 1 for references) and many biochemical assays, including ours, measure the GLT-1-sensitive component of uptake through the use of DHK. Therefore, it is possible that we were unable to see an impairment in the overall glutamate clearance in *YAC128* striatum as a result of compensation by non GLT-1 transporters, including GLAST or EAAC1 (ref. 24). To assess the contribution of GLT-1 to total glutamate clearance, we evoked glutamate release as above, and measured iGluSnFR fluorescence decay in the absence and then presence of increasing concentrations of DHK. We found that 500 μM DHK did not influence the decay tau following the application of 250 μM DHK; therefore, 250 μM was used as a saturating concentration. As expected, DHK increased the decay tau of iGluSnFR signals in both WT (*FVB/N*) and *YAC128* striatum (Fig. 3a–c). However, the relative increase in iGluSnFR decay tau was similar for both the genotypes, suggestive of a similar GLT-1-mediated role in glutamate clearance. When the non-selective glutamate transporter inhibitor threo-β-benzyloxyaspartic acid (TBOA; 100 μM, ref. 25) was bath applied for 10 min to block all transporters, we observed a much larger increase in the decay tau in comparison with GLT-1-specific blockade. Again this effect was similar for both genotypes (Fig. 3a,d). Background fluorescence also increased in response to transporter blockade, likely the consequence of an increase in ambient extracellular glutamate^26^, and occurred equally in both genotypes (Supplementary Fig. 5a). Thus, when measuring the extracellular glutamate dynamics in a system with preserved synaptic structure, we see no evidence supporting an impairment of transporter-mediated glutamate uptake in the HD striatum.

As it is possible that the temporal profiles within the microenvironments immediately adjacent to the neuronal and astrocytic surface membranes may differ, we also repeated some experiments in 2-month-old WT (*FVB/N*) and *YAC128* mice injected with iGluSnFR under the control of the astrocyte-specific GFAP promoter. For these experiments, the mice were injected intrastriatally with AAV2/1-GFAP-iGluSnFR, and the experiments were carried out as before. Again, we saw no evidence of impaired glutamate clearance following synaptic release evoked by single pulses or by HFS trains (50 pulses, 100 Hz, 200 μA; Supplementary Fig. 6a–d). As observed for synapsin-iGluSnFR, the application of DHK and TBOA failed to reveal a genotype difference in the GLT-1 and non GLT-1-mediated components of glutamate clearance (Supplementary Fig. 6e), and again, no differences in the DHK- or TBOA-induced increases in background fluorescence were observed (Supplementary Fig. 5b). We also noted a lack of correlation between the response peak and decay tau within a given stimulation paradigm (Supplementary Fig. 6f,g), suggesting that our results were not influenced by the magnitude of glutamate release.

**Figure 4:**
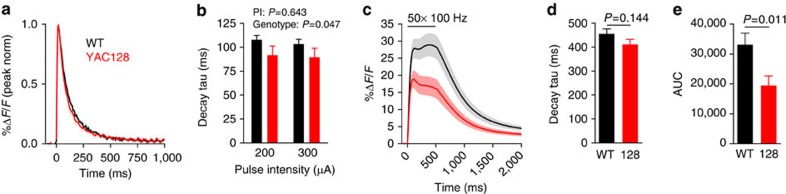
Striatal neurons are exposed to lower levels of synaptically released glutamate in 15-month-old *YAC128* mice. (**a**) Grouped data showing the average iGluSnFR response (±s.e.m.) to a single pulse (200 μA) in the dorsal striatum from WT (*FVB/N*; black) and age-matched, *YAC128* (red) mice. Responses are normalized (norm) to their peaks for direct comparisons of decay time course. (**b**) Bar graph showing iGluSnFR decay in response to single pulses at different pulse intensities (PI; WT *n*=9 per bar; *YAC128 n*=8 per bar; RM two-way ANOVA). (**c**) Average iGluSnFR response to a high-frequency stimulation (HFS) consisting of 50 pulses over 500 ms (200 μA). The raw data are shown to highlight the dramatic difference in the response size between WT (*n*=20) and *YAC128* (*n*=21) under these conditions. (**d**) Bar graph showing the decay tau from the experiments in **c** (unpaired *t*-test). (**e**) Bar graph showing the area under the curve (AUC) from the experiments shown in **c** (unpaired *t*-test).

It is possible that the previously reported uptake impairment in HD is progressive, with a greater dysfunction observed at later disease stages. Thus, we asked whether a slower clearance of synaptically released glutamate could be observed in *YAC128* mice well after the onset of an overt motor phenotype^27^. To test this, we aged animals to approximately 14 months (Supplementary Table 1) before the intrastriatal injection of AAV2/1-synapsin-iGluSnFR. All the experiments from this group were performed at room temperature to ensure optimal slice viability from the aged animals. On a single pulse (200 and 300 μA), we saw no significant genotype differences in the response peak (200 μA; WT: 6.4±2.5%Δ*F*/*F*, *YAC128*: 6.3±2.7%Δ*F*/*F*; 300 μA; WT: 9.1±3.1%Δ*F*/*F*, *YAC128*: 9.0±3.6%Δ*F*/*F*, WT *n*=9, *YAC128 n*=8, *P*=0.96, repeated-measures (RM) two-way analysis of variance (ANOVA)). However, in stark contrast to our original hypothesis, we observed a small but significant acceleration of iGluSnFR decay following single pulses (Fig. 4a,b). When slices were challenged with HFS (50 pulses, 100 Hz, 200 μA), there was a clear reduction in the peak response from *YAC128* slices (Fig. 4c), in agreement with a loss of excitatory synapses and a reduction in glutamate release in late-stage HD striatum^28^,^29^. Although there was no significant difference in the decay tau between the two genotypes under these conditions (Fig. 4d), there was a decrease in the area under the curve of evoked iGluSnFR responses (Fig. 4e), suggesting that the total amount of glutamate sensed at the surface of neurons is lower in aged *YAC128* mice. As with our experiments in younger *YAC128* mice, we observed no evidence to support a deficit in glutamate clearance.

### *In situ* glutamate clearance in *R6/2* HD mice

**Figure 5:**
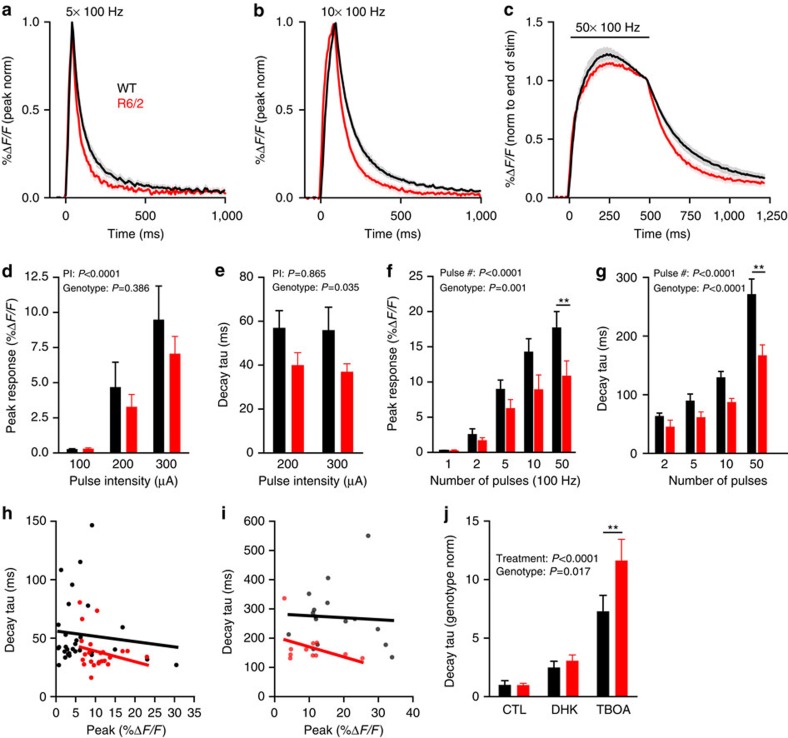
Extracellular glutamate clearance is accelerated in the *R6/2* model of HD at 10 weeks of age. (**a**–**c**) Grouped data showing the average iGluSnFR response (±s.e.m.) to 5 (**a**), 10 (**b**) and 50 (**c**) pulses (100 Hz, 100 μA) in the dorsal striatum from 2-month-old WT (*B6CBAF1/J*; black) and age-matched, *R6/2* (red) mice. The responses are normalized (norm) to their peaks to simplify comparisons of decay time course. (**d**,**e**) Bar graphs of response size (**d**) and decay (**e**) to single pulses of varying pulse intensities (PI). WT *n*=16 per bar, *R6/2 n*=11, 12, 12 for 100, 200, 300 μA, respectively, two-way ANOVA. (**f**,**g**) Bar graphs of response size (**f**) and decay (**g**) to an increasing number of pulses with 10 ms interpulse intervals. WT *n*=15, 15, 16, 16, 16 for 1, 2, 5, 10, 50 pulses, respectively, *R6/2 n*=11 per bar, two-way ANOVA; **P<0.01 Bonferroni *post hoc* comparison. (**h**,**i**) Linear regression plots showing no correlation for either WT (black) or *R6/2* (red) between iGluSnFR response size and decay tau following a single pulse at 200 μA (**h**; WT *r*^2^=0.011, *P*=0.575; *R6/2 r*^2^=0.059, *P*=0.264) or 50 pulses at 100 Hz (100 μA, **i**; WT *r*^2^=0.049, *P*=0.829, *R6/2 r*^2^=0.170, *P*=0.208). (**j**) Bar graph showing the fold-increase in iGluSnFR decay kinetics following a 10-min bath application of the GLT-1-specific blocker dihydrokainic acid (DHK; 250 μM) or the non-selective glutamate transporter inhibitor threo-β-benzyloxyaspartic acid (TBOA; 100 μM). The values are normalized to the genotype average in baseline (untreated) conditions. WT *n*=8 per bar; *R6/2 n*=6, 6, 5 from left to right; two-way ANOVA. **P<0.01 Bonferroni *post hoc* comparison.

Biochemical quantification of glutamate uptake in synaptosomes as well as no-net-flux microdialysis experiments have also strongly suggested an uptake impairment in the *R6/2* mouse model of HD (Table 1). This transgenic model, which expresses a toxic amino (N)-terminal fragment of the mutant huntingtin protein, is particularly aggressive and shows an HD-like phenotype starting as early as 1 month of age^30^. To assess the extracellular glutamate clearance following synaptic release in this model of HD, we injected 4–5-week-old *R6/2* and age-matched WT (*B6CBAF1/J*) mice with AAV2/1-synapsin-iGluSnFR and imaged evoked responses 5–6 weeks later when they were approximately 10 weeks of age (Supplementary Table 1). To help ensure that we were not missing any potential genotype difference based on our stimulus parameters, we conducted a more extensive input–output profile of evoked iGluSnFR responses by varying both the stimulus number (at 100 Hz) and the intensity of a single pulse (Fig. 5a–c). Consistent with the loss of excitatory inputs to the striatum in this model^31^,^32^, iGluSnFR responses were generally smaller in the *R6/2* mice (Fig. 5f). In both the genotypes, iGluSnFR peaks were dependent on both the stimulus intensity and the number of stimuli, whereas the decay was dependent on the number of stimuli but not the intensity (Fig. 5d–g). As we saw some evidence for in *YAC128* mice, iGluSnFR decay taus were found to be significantly faster in *R6/2* mice (Fig. 5e,g). This effect cannot be explained by the reduced response sizes in *R6/2* mice as there was no significant correlation between peak and decay within a constant stimulation condition (Fig. 5h,i). This effect is also not explained by differences in iGluSnFR expression as there was no correlation between expression level and decay kinetics (Supplementary Fig. 7). We also observed no significant genotype difference in the decay of iGluSnFR intensity with increasing distance from the central region of maximal release (Supplementary Fig. 8).

When we assessed the relative contribution of GLT-1 and non-GLT-1-mediated uptake to the decay of iGluSnFR responses, we found that a saturating concentration of DHK (250 μM) increased the decay tau to the same extent in both genotypes. However, we observed a significantly larger increase in decay tau in the *R6/2* mice upon addition of 100 μM TBOA to block all transporter-mediated uptake (Fig. 5j). The increases in basal fluorescence were not significantly different between the genotypes (Supplementary Fig. 5c). These pharmacological data suggest that the accelerated glutamate clearance observed under basal conditions in the *R6/2* striatum may be a result of enhanced transporter-mediated uptake. As DHK alone affected both genotypes equally, we conclude that the enhanced uptake in *R6/2* striatum is likely to be mediated by a transporter other than GLT-1.

**Figure 6:**
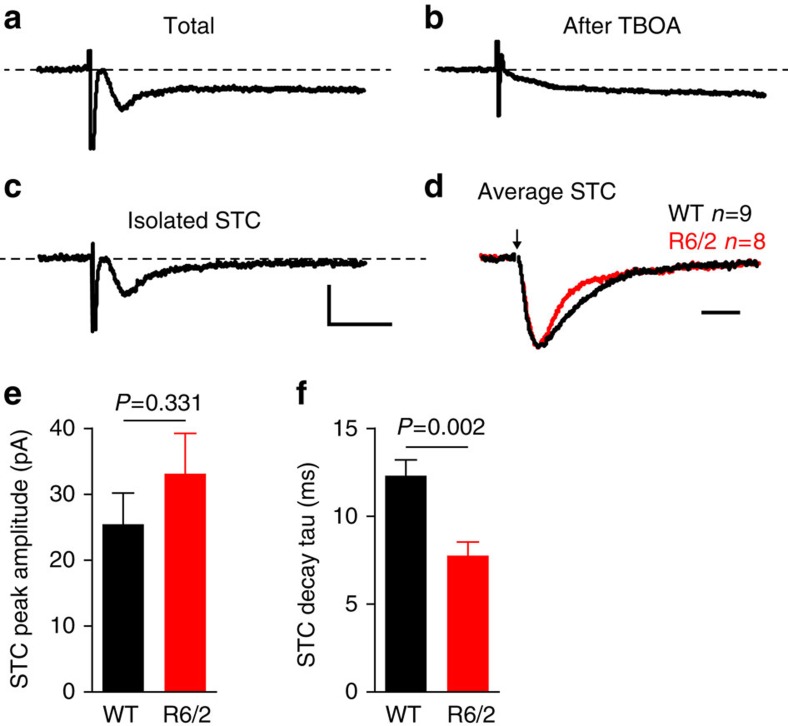
Synaptically activated currents decay faster in *R6/2* astrocytes. (**a**) Representative synaptically activated transporter current (STC) recorded in a striatal astrocyte, evoked by local electrical stimulation of glutamatergic afferents. The isolated STC (**c**) was obtained by subtracting the response in the presence of TBOA (100 μM, **b**) from that before TBOA application (**a**). Scale bars, 20 pA, 20 ms. (**d**) Average of all the isolated STCs recorded from WT (black, *n*=9) and *R6/2* (red, *n*=8) astrocytes. The peaks are scaled for direct comparisons of the decay kinetics. Scale bar, 10 ms (**e**) Bar graph showing no difference in the average size of the isolated STCs (unpaired *t*-test). (**f**) Bar graph showing a significant reduction in the decay time of STCs recorded from *R6/2* astrocytes (unpaired *t*-test).

The observed acceleration in synaptically evoked glutamate clearance in the *R6/2* striatum was unexpected as it contrasts with the conclusions drawn from previous work and opposes general working models of the neurobiology of HD. Thus, we employed an additional technique that can be used to quantify relative changes in glutamate clearance *in situ* following synaptic release^25^. Glutamate uptake is electrogenic, and the associated cation current can be recorded from astrocytes using conventional whole-cell patch clamp techniques. We patched SR101-labelled astrocytes in the striatum from WT (*B6CBAF1/J*) and *R6/2* mice at approximately 10 weeks of age; an age at which clear impairments in transporter-mediated glutamate uptake are detected by biochemical assays (Table 1). A passive response to both positive and negative current injections^33^ confirmed each recorded cell's identity as an astrocyte (data not shown). Basic membrane properties recorded from both the genotypes are shown in Table 2. Glutamate release was evoked by a glass stimulating electrode placed 200–250 μm dorsal to the recorded astrocyte. Synaptically evoked transporter currents (STCs) were isolated by subtracting the evoked current in the presence of TBOA from that before TBOA application (Fig. 6a–c). Varying stimulation intensities were required to elicit a robust STC in each slice (see ‘Methods' section), and there was no significant difference in peak amplitudes of the STCs evoked for each genotype (Fig. 6e). In agreement with our imaging data, we observed a significant acceleration of the decay of STCs recorded from *R6/2* astrocytes compared with age-matched controls (Fig. 6d,f). The membrane resistance and capacitance were also found to be significantly different between the genotypes, although there was no significant correlation between either of these parameters and STC decay (*r*^2^=0.033, *P*=0.640 for membrane resistance and STC decay; *r*^2^=.0123, *P*=0.358 for membrane capacitance and STC decay), suggesting that the differences observed in STC decay cannot be accounted for by differences in these membrane properties.

## Discussion

On the basis of prior studies, a widely held view has emerged in the HD literature—that impaired glutamate uptake plays a key role in the associated neurodegeneration. Although glutamate clearance in HD had not been tested until now in the brain slice, with intact neuronal circuits and synapses, this view has rarely been questioned and is frequently mentioned in the literature as a key mechanism of disease pathogenesis. Here, we asked whether the clearance of synaptically released glutamate is slower in the HD striatum by using real-time, *in situ* measures of extracellular glutamate dynamics. To our surprise, we found no evidence of impaired glutamate uptake. In stark contrast, we observed a significant acceleration of glutamate clearance under certain experimental conditions, particularly in the *R6/2* model of HD. Our data suggest that glutamate transporter dysfunction is not a major component of HD pathology and calls for a revision of our current model of synaptic dysfunction and contributors to excitotoxicity in this disease. Moreover, our results cast doubt on the potential use of glutamate uptake enhancement as a therapeutic strategy for the treatment of HD.

By far, the most common approach to quantify glutamate uptake in brain tissue is by liquid scintillation counting of radio-labelled glutamate or aspartate that is taken up by a synaptosomal preparation over a period of minutes. STC recording *in situ* has also been used to great effect; however, this technique is time consuming, technically demanding and is only routinely performed by a relatively small number of laboratories. The last few years have seen a rapid rise in the availability of optogenetic sensors, and with the introduction of iGluSnFR, a recent study has generated and characterized a sensitive and specific single-wavelength glutamate sensor with rapid on–off kinetics^23^. We show here that this sensor is highly sensitive to the relative differences in glutamate clearance capacity. The characteristics of iGluSnFR provide this approach with a number of advantages over standard biochemical uptake assays when quantifying the differences in glutamate clearance capacity. First, it allows for the comparison of glutamate dynamics *in situ*. Glutamate transporters are located on both neurons and glia and are generally found at a higher concentration near synaptic sites^34^,^35^. The rapid release from presynaptic terminals and reuptake by nearby transporters characterizes synaptic transmission, and both of these obligatory features are lost with biochemical uptake assays. Moreover, in synaptosomes, exogenous substrate appears to be predominantly taken up by GLT-1 located on the nerve terminals rather than astrocytes, despite the relatively negligible physiological importance of neuronal GLT-1 compared with astrocytic GLT-1 (refs 21, 22). These results question the precise physiological relevance of uptake assays that utilize exogenous glutamate or aspartate. Second, the time course of evoked iGluSnFR responses reflects not only transporter-mediated uptake but also diffusion, permitting an overall measure of glutamate clearance. Third, as a genetically encoded sensor, iGluSnFR expression can be driven under the control of a specific promoter. This allowed us to test a leading hypothesis in HD excitotoxicity by comparing the dynamics of glutamate sensed at the neuronal versus astrocytic extracellular surface.

On the other hand, imaging glutamate requires an exogenous glutamate binding site to be introduced into the brain, resulting in artificial competition with the natural time course of glutamate binding and unbinding to transporters and receptors. However, competitive glutamate receptor blockade did not alter the iGluSnFR decay time course, suggesting a negligible buffering effect by ionotropic glutamate receptors in these experiments. Thus, it is unlikely that the increase in surface NMDA receptors in the HD striatum^36^ influenced our measurements of iGluSnFR decay. There also appeared to be no significant correlation between iGluSnFR expression and decay tau, at least within the range of sensor expression located at the stimulation sites. Moreover, despite a significant difference in the magnitude of the glutamate decay time course measured by iGluSnFR imaging versus synaptically activated transporter currents recorded from astrocytes, relative differences in decay tau were preserved between the genotypes using the two methods.

In hindsight, perhaps the well-established notion of an uptake dysfunction in HD was unfounded. For example, a glutamate uptake impairment would be expected to increase the basal levels of extracellular glutamate in the striatum^37^,^38^, as demonstrated by the observed increase in basal (unstimulated) iGluSnFR intensity^26^ in response to glutamate transporter inhibition in the present study. However, this has not been reported in studies using microdialysis; in some cases, no differences in basal extracellular glutamate were detected in the *R6/2* striatum^16^,^18^,^39^, while in others, a reduction was noted in both the *R6/1* and *R6/2* models of HD^40^,^41^. Furthermore, glutamate uptake blockade significantly slows the decay of NMDAR-mediated excitatory postsynaptic currents (EPSCs) recorded from the striatal spiny projection neurons, yet basal differences between the WT and *YAC128* NMDAR EPSC decay kinetics are not readily detected^36^. Interestingly, EAAC1-mediated uptake is enhanced in a cellular model of HD^42^, consistent with our findings of accelerated uptake, which were particularly evident in the *R6/2* model. Combined with the known reduction of functional excitatory synapses and release probability in late-stage HD^43^, it is likely that striatal neurons are exposed to less glutamate overall, particularly in late-stage HD. This is demonstrated here by the reduction in the area under the curve of iGluSnFR signals in aged HD mice. In sum, we conclude that while the biochemical data (see Table 1) may suggest an impairment in GLT-1-mediated uptake into nerve terminals^21^,^22^, our data clearly demonstrate that there is no deficit in the overall clearance of synaptically released glutamate in the HD striatum. There also appears to be no morphological evidence suggesting an impairment in extracellular glutamate diffusion; the number of tripartite synapses as well as the average synapse-to-astrocyte distance are similar in the WT and *YAC128* striatum at 1 year of age^36^.

It was recently reported that depolarized astrocytes and poor extracellular potassium buffering are evident in late-stage HD mouse models, including the *R6/2* mice^44^. It is known that astrocyte depolarization can impair transporter-mediated uptake^45^, which is opposite to our findings of enhanced glutamate clearance in the *R6/2* striatum. Moreover, in the present study, we did not observe a significant difference in the resting membrane potential of striatal astrocytes recorded from the *R6/2* mice at approximately 10 weeks of age, contrasting with this recent finding^44^. The reason for this discrepancy remains unclear, but may involve differences in the composition of the intra- and extracellular solutions. Nonetheless, our data regarding extracellular glutamate clearance in the HD mice were consistent regardless of whether we measured glutamate clearance with STC recordings, in which astrocytes are voltage clamped, or with iGluSnFR, in which astrocytes are left unperturbed.

If there is no uptake deficit in HD, then why would ceftriaxone, a beta-lactam antibiotic that increases GLT-1 expression, have a beneficial effect on the *R6/2* HD phenotype^18^? One possibility is that increasing GLT-1 expression, even in the absence of an uptake deficit, would limit the activation of cell death-associated extrasynaptic NMDA receptors, which are upregulated in early-stage HD^7^,^36^. Another possibility, and one proposed by a recent study showing no exacerbation of the HD phenotype in the *R6/2* mice that were also heterozygous for the gene encoding GLT-1 (ref. 46), is that ceftriaxone's beneficial effect may be independent of GLT-1 expression and rather arise from its known effect on various cell-survival transcription factors^47^,^48^. From the point of view of a treatment for HD patients, enhancing uptake in the absence of a deficit is likely to result in unwanted side effects by interfering with physiological processes that depend upon glutamate spill-over^49^,^50^. Indeed, ceftriaxone has been shown to impair various forms of plasticity, as well as hippocampal-dependent learning, when administered to WT animals^51^,^52^. In light of these findings as well as those of the present study, we propose that a more effective therapeutic strategy for the prevention of excitotoxic cell death in HD may be to block extrasynaptic NMDA receptors or their downstream signalling^7^. Alternatively, studies providing insight into the precise mechanism of ceftriaxone's protective effect in HD may uncover novel therapeutic avenues.

The data presented here oppose a well-accepted view of HD pathogenesis. We argue that the imaging and electrophysiological tools used here allow for a more physiologically relevant quantification of extracellular glutamate dynamics than previous measures of glutamate uptake in HD. On the basis of our findings, we propose that a deficit in glutamate clearance is unlikely to be a major contributor to excitotoxic cell death in HD and that therapeutic strategies should be revised, if necessary, as a result. We propose that excitotoxicity in HD is not a result of a double-edged sword consisting of the combination of elevated extrasynaptic NMDA receptors and impaired glutamate uptake. Despite the lack of a glutamate clearance deficit, robust extracellular glutamate signals, lasting for tens to hundreds of milliseconds, were indeed detected in both HD mouse models under a variety of physiologically relevant stimulation conditions, suggesting that extrasynaptic NMDA receptors do not require uptake impairments to become activated. Thus, these two different pathological mechanisms do not need to occur in tandem for a detrimental effect to ensue. On a broader scale, our data also demonstrate a lack of correlation between the results obtained from biochemical measures of glutamate uptake and real-time measurements of glutamate clearance *in situ* using optogenetic reporting and electrophysiology. The sensitivity of iGluSnFR to relative differences in glutamate clearance capacity suggests that this will prove to be an invaluable tool to the future study of glutamate transporters in both health and disease.

## Methods

### Animals

All the procedures were carried out in accordance with the Canadian Council on Animal Care and approved by the University of British Columbia Committee on Animal Care (Protocol A11-0012). The animals were housed on a 12 h:12 h light:dark cycle and had *ad libitum* access to standard lab chow and water. A total of 60 male mice were used in this study. The ages used for each set of experiments is summarized in Supplementary Table 1. Data from the *YAC128* model were compared with age-matched control mice of the same background strain (*FVB/N*), while data from the *R6/2* model were compared with their background strain, *B6CBAF1/J*. The *FVB/N* and *YAC128* (line 55) mice were bred on site. The *B6CBAF1/J* (stock 100011) and *R6/2* mice (stock 002810) were purchased from The Jackson Laboratory. In the manuscript, the term ‘wild-type' (WT) is used to refer to control mice; either *FVB/N* (for comparisons with *YAC128* mice) or *B6CBAF1/J* (for comparisons with *R6/2* mice). A recent review provides a detailed description of the various mouse models of HD, including the *YAC128* and *R6/2* models^53^.

### Virus injection and brain slice preparation

The mice were anaesthetized with isoflurane inhalation (2%) and maintained on 1.5% isoflurane for the duration of the surgical procedure. The body temperature was held constant at 37±1 °C with a heating pad. Then 1 μl of AAV2/1.hSyn.iGluSnFr.WPRE.SV40 or AAV2/1.GFAP.iGluSnFr.WPRE.SV40 (Penn Vector; provided by Dr Loren Looger, Janelia Farm Research Campus of the Howard Hughes Medical Institute) was injected into the dorsal striatum through a glass pipette attached to a Hamilton syringe and the injection rate was controlled over a 10-min period through the use of a micro-syringe pump (UMC4; World Precision Instruments). Stereotaxic co-ordinates with respect to Bregma were (in mm): 0.75 anterior, 2.0 lateral, 2.5 ventral. Approximately 1 month following the viral injections, the mice were anaesthetized with isoflurane, decapitated and the coronal brain slices (400 μm) containing the striatum were obtained as described previously^54^.

### iGluSnFR imaging

The slices were transferred to a standard submerged recording chamber through which oxygenated ACSF^54^ (pH=7.3±0.1) was perfused at a flow rate of 2–3 ml per min at room temperature or at 32 °C±1 °C, as indicated. The ACSF consisted of (in mM): 125 NaCl, 2.5 KCl, 25 NaHCO_3_, 1.25 NaH_2_PO_4_, 1 MgCl_2_, 2 CaCl_2_, 10 glucose. A 0.1 MΩ resistance monopolar tungsten stimulating electrode (Microprobes; tip diameter 2–3 μm) was positioned in the dorsal striatum at a depth of 50–100 μm, and electrical stimulation parameters were controlled by an A-M systems isolated pulse stimulator (Model 2100) and a World Precision Instruments stimulus isolator (A385). The stimulus duration was 0.1 ms. iGluSnFR fluorescence was excited by a 470 nm LED and visualized with a CCD camera (1M60, Pantera, Dalsa) coupled to a pair of back-to-back photographic lenses (50 mm, 1.4f; 135 mm, 2.8f) and a 530 nm bandpass filter. For each slice, the LED intensity was manually adjusted to generate a basal fluorescence level between 1,000 and 2,000 arbitrary units (12-bit scale). An 8 × 8 pixel binning was used to allow an image acquisition rate of 150 Hz (128 × 128 pixels per image). Clampex software was used to trigger LED activation and electrical stimulation, and images were acquired with XCAP software (EPIX, Inc.). For each experiment, the responses were evoked every 30 s for 7 min, with every third trial a blank trial without stimulation in order to enable subtraction of any bleaching artifact. In five randomly selected experiments from five different injections, 1,440 ms of LED exposure resulted in a 5.74±1.03%Δ*F*/*F* reduction in basal, unstimulated iGluSnFR fluorescence during that time period; all of which fully recovered before the start of the next trial 30 s later. The responses were averaged over the 10 stimulation trials and normalized to the basal fluorescence obtained from the five blank trials so that the LED-induced reduction in iGluSnFR intensity did not affect our measurements of decay kinetics in the evoked responses. The magnitude of all evoked iGluSnFR data are expressed as a percentage change in fluorescence above basal fluorescence and are denoted as %Δ*F*/*F*. Endogenous fluorescence^55^, was of little concern in the present study: a low-level fluorescence with kinetics much slower than that of iGluSnFR responses was observed only after prolonged HFS in the absence of TTX (50 pulses, 100 Hz, 200 μA; Supplementary Fig. 1) and not following single pulses or shorter bursts. Endogenous fluorescence was assessed by subjecting a brain slice from a non-iGluSnFR-injected *FVB/N* mouse to the same conditions as those used for iGluSnFR experiments.

To image iGluSnFR responses to exogenous glutamate, increasing concentrations of glutamate were supplied to the bath while iGluSnFR was illuminated with low LED intensity and imaged at 1 Hz. The glutamate was increased from (in μM) 0, 1, 10, 100, 300 to 1,000, and held ‘clamped' at each concentration for a minimum of 5 min. To prevent ongoing uptake of the exogenous glutamate, 100 μM TBOA was co-applied throughout. To minimize excitotoxic damage and the effects of exogenous glutamate, TTX (1 μM), kynurenic acid (1 mM) and LY341495 (30 μM) were also bath applied throughout.

### Primary neuronal co-cultures and immunofluorescence

Cortico-striatal co-cultures were prepared from E17–18 striatal and cortical tissues, as described previously^54^,^56^, although striatal neurons were not GFP-transfected on the day of plating in the present study. The cells were grown on poly-D-lysine-coated glass coverslips in 24-well plates with 1 ml of plating medium (Neurobasal medium, B27, glutamine and penicillin/streptomycin; Invitrogen) per well. On DIV4, 0.1 μl of the AAV2/1.hSyn.iGluSnFr.WPRE.SV40 virus (2.56 × 10^13^ GC per ml) was added to each well. On DIV7, the media were removed and replaced with 1 ml fresh plating media without virus, and subsequent half media changes occurred every 3 days thereafter. On DIV15-16, surface iGluSnFR was amplified by live staining with a chicken anti-GFP antibody (1:1,000; Abcam ab13970) for 10 min at 37 °C before fixation in 4% paraformaldehyde/sucrose for 10 min. Following fixation, the cells were washed in phosphate-buffered saline (PBS) before incubation with anti-chicken Alexa 568 secondary antibody (1:1,000; Invitrogen A11041, 1 h at room temperature). The cells were then washed and permeabilized with PBS containing 0.03% Triton X-100 (PBST). The coverslips were then exposed to a DARPP32 primary antibody (rabbit anti-DARPP32; #2306, Cell Signaling Technology; 1:1,000; dissolved in PBST containing 2% normal goat serum) for 1 h at room temperature to help identify the striatal spiny projection neurons ; morphological criteria were also used^57^. An AMCA-conjugated anti-rabbit secondary antibody was used to visualize DARPP32 (1:50; Jackson ImmunoResearch 711-155-152). The images were acquired with a Ziess Axiovert 200 M as described previously^54^. ImageJ was used to draw three ROIs around the dendrites for each DARRP-32-positive cell analysed. The average fluorescence intensity for the three ROIs for each of the red and green channels (representing surface and total iGluSnFR, respectively) was calculated and a ratio of surface:total staining was generated for each cell. The number of cells analysed is given as *N*, from three different batches each of *YAC128* and WT co-cultures.

### Visualizing iGluSnFR in single cells in fixed brain slice

To visualize iGluSnFR expression in single cells *in vivo*, we used a dilution strategy^58^ that avoids the high-density expression that we normally achieve with high titre iGluSnFR injections. We co-injected a mixture of diluted AAV2/1.hSyn.Cre.WPRE.hGH (1.46 × 10^9^ GC per ml; Penn Vector Core) and Cre-dependent iGluSnFR virus (AAV2/1.hSyn.Flex.iGluSnFR.WPRE; Penn Vector; provided by Dr. Loren Looger, Janelia Farm Research Campus of the Howard Hughes Medical Institute). Approximately 3–4 weeks following the injection, the *FVB/N* and *YAC128* mice were transcardially perfused with a 4% solution of paraformaldehyde in PBS. The perfused brains were post-fixed in 4% paraformaldehyde for 24 h, and then submerged in graded sucrose concentrations (24 h each at 10, 20 and 30% sucrose). The brains were embedded with O.C.T. compound (Sakura) and rapidly frozen in the bottom of the Cryomold, then sectioned at 18–20 μm on a cryostat (ThermoScientific Micron HM525) and submerged in PBS. The sections were then incubated in blocking solution (2.5% BSA, 0.1% Triton X-100, 0.02% sodium azide in PBS) for 45 min, followed by overnight 4 °C incubation in chicken anti-GFP primary antibody (1:500; Abcam ab13970) diluted in blocking solution. Following washing with PBST, the sections were incubated with anti-chicken Alexa488 secondary antibody (1:500 in blocking solution for 1 h at room temperature), washed and then slide-mounted with Fluoromount. The images were acquired with a Leica SP8 confocal microscope with a white light laser and hybrid photomultiplier detector with a × 63 oil immersion objective.

### Pharmacology

All the drugs were purchased from Tocris with the exception of tetrodotoxin (TTX), which was purchased from Affix Scientific. The cocktail of antagonists added to the ACSF for select experiments (described in the ‘Results' section) were as follows: picrotoxin (100 μM), D-APV (50 μM), DNQX (10 μM), sulpiride (10 μM), SCH 23390 (4 μM), scopolamine (20 μM), hexamethonium bromide (100 μM), CGP 52432 (1 μM) and LY 341495 (3 μM). This cocktail of drugs was used to block GABA_A_, NMDA, AMPA, D2-like and D1-like dopamine, muscarinic and nicotinic acetylcholine, GABA_B_ and metabotropic glutamate receptors, respectively. All the drugs, including transporter inhibitors DHK and TBOA, were bath applied for at least 10 min. The saturating effect of 250 μM DHK on GLT-1 inhibition was confirmed by a lack of further effect of 500 μM DHK on iGluSnFR decay time course (not shown).

### iGluSnFR analysis

Evoked iGluSnFR responses were analysed in ImageJ by placing a 4 × 4 pixel (93.8 × 93.8 μm) ROI at the position where glutamate release was maximal, typically immediately adjacent to the stimulating electrode. All the quantifications, including peak fluorescence intensity, were obtained in ImageJ by averaging the iGluSnFR intensity within this 4 × 4 pixel region for each frame of a given response. When we quantified iGluSnFR dynamics outside of this central region of release, a 4 × 4 ROI was made 500 μm ventral to the region of greatest iGluSnFR peak intensity. All decay kinetics of evoked iGluSnFR responses were quantified through the use of a nonlinear regression single-exponential curve fit performed in GraphPad Prism. The curve fitting was applied from the peak of the response to the final frame of acquisition in all cases of single pulses and short bursts. For HFS trains consisting of 50 pulses at 100 Hz, curve fitting was applied immediately after stimulation offset until the end of acquisition. For presentation purposes in the figures, many traces were normalized to their peak value to allow for easier comparisons of the decay kinetics between groups; however, in such instances, all corresponding raw traces have also been provided as Supplementary Figs 9–13. To calculate the spread of iGluSnFR signals, all frames of a given response were *z*-projected as a single maximum intensity image and 585 μm (25 pixel) lines were drawn in the medial, lateral and ventral directions, originating from the pixel of highest intensity. iGluSnFR intensity values were calculated along the length of these lines in ImageJ and the resulting values were normalized to the pixel of greatest intensity. To measure iGluSnFR responses in WT and *YAC128* striatal slices to exogenous glutamate, a ROI was drawn in the dorsal striatum around the extent of iGluSnFR expression and average pixel intensities within the ROI were calculated for each frame. For each glutamate concentration, the intensity values from a total of 11 frames, centred on the peak response for that concentration, were averaged and plotted. The data were calculated against the baseline value in the presence of TTX, TBOA, kynurenic acid and LY341495, before the addition of 1 μM glutamate.

### Electrophysiology

The non-injected hemisphere from *B6CBAF1/J* controls or *R6/2* HD mice was used for electrophysiological recordings. The slices were obtained as described above. After recovery, SR101 was added to the holding chamber for a minimum of 30 min to label astrocytes before transferring a hemisection to a recording chamber perfused with ACSF for 1–2 ml per min at a temperature of 32±1 °C. Astrocytes, identified by SR101 uptake, were patched with pipettes containing an intracellular solution that consisted of (in mM): 145 potassium gluconate, 1 MgCl_2_, 10 HEPES, 1 EGTA, 2 MgATP and 0.5 Na2GTP. Astrocytes at the surface of the slice were avoided in these experiments and only those deep in the tissue were targeted for STC recording. Upon successful break-in, the astrocyte phenotype was confirmed by their passive response to negative and positive current injection^33^. Synaptically activated transporter currents were evoked by single pulses through a glass stimulating electrode placed 150–200 μm dorsal to the recorded astrocyte. Responses were low-pass filtered at 1 kHz. From one slice to the next, the amount of current required to generate a measurable STC varied; thus, stimulus intensity was adjusted accordingly for each slice. The average current used to evoke an STC in these experiments did not differ between genotypes (WT: 362.5±60.3 μA, *n*=9; *R6/2*: 375.0±18.9 μA, *n*=8, *P*=0.85, *t*-test). An STC was evoked every 20 s. After establishing a baseline, TBOA (100 μM) was bath applied for 7–10 min. Pure STCs were isolated by subtracting the average of at least five responses in the presence of TBOA from that before TBOA. As astrocytes were prone to gradual increases in series resistance in our hands, we tolerated an increase in the series resistance up to a maximum of 35% from the baseline period to the time TBOA exerted its effect. Importantly, there was no genotype difference in the series resistance increase during this time (WT: 10.1±5.8%, *n*=9; *R6/2*: 19.1±3.6%, *n*=8, *P*=0.24, *t*-test).

### Glutamate uptake assay

The glutamate uptake in synaptosomes from the brain tissue was measured as previously described^17^. The striatum was dissected from 1- and 3-month-old WT *FVB/N* and *YAC128* mice and homogenized by mashing tissue in 1.4 ml ice-cold homogenizing buffer (5 mM Tris, 320 mM sucrose, 5 mM EDTA, 5 mM EGTA, 1 mM sodium orthovanadate, protease inhibitors). The nuclei and debris were pelleted at 14,000*g* at 4 °C for 10 min. The pellet was vortexed and washed twice with 1 ml cold homogenizing buffer, then resuspended in 300 μl tissue buffer (5 mM Tris, 320 mM sucrose). The suspension was divided into three equal parts (100 μl each tube) and was washed with 1 ml cold tissue buffer and spun down at 14,000*g* at 4 °C for 10 min. The pellets in each tube were resuspended in 250 μl Na^+^-free Krebs buffer (120 mM choline chloride, 25 mM Tris, 5 mM KCl, 1 mM KH_2_PO_4_, 1 mM MgSO_4_, 10 g D-glucose, 2 mM CaCl_2_), Na^+^-plus Krebs buffer (120 mM NaCl, 25 mM Tris, 5 mM KCl, 1 mM KH_2_PO_4_, 1 mM MgSO_4_, 10 g D-glucose, 2 mM CaCl_2_) and DHK/Na^+^-plus Krebs buffer (500 μM DHK in Na^+^-plus Krebs buffer) respectively, as previously described (Rothstein *et al*., 1992). All the samples were pre-incubated for 10 min at 37 °C and returned onto cold ice. Each condition had duplicate tubes with 100 μl homogenate in each tube, and 50 μl was reserved for protein concentration determination. A 25 μl of ^3^H-labelled glutamate (PerkinElmer) mix (0.05 mM L-glutamate with 5μCi ^3^H glutamate in appropriate Krebs) was added to each sample (Na^+^- glutamate mix for Na^+^ plus samples, DHK-glutamate mix for DHK/Na^+^ plus samples and Na^+^-free glutamate mix for Na^+^-free samples). The samples were then incubated for 4 min in a 37 °C water bath. The uptake was stopped by quickly placing the tube back in iced water. The removal of the unincorporated radiolabelled glutamate was accomplished by centrifugation for 5 min at 10,000 r.p.m. The pellet was washed three times with 400 μl of cold-tissue buffer followed by centrifugation. Triton (0.8%) was added to the pellets. The radioactivity was assessed by performing a liquid scintillation count, and protein concentration was determined by BCA protein assay kit.

### Statistics

All the data are presented as mean±s.e.m and normality was formally assessed in Graphpad prism. As all the data were determined to be normally distributed, parametric statistics were used throughout. The statistical tests used include: *t*-test, paired *t*-test, two-way ANOVA and RM two-way ANOVA. All the *t*-tests were performed as two-tailed *t*-tests. RM two-way ANOVA was only used when all the data within a data set for a given factor were paired. For data sets containing both paired and unpaired data, two-way ANOVA was used. The statistical test used for each experiment is stated in the text and/or corresponding figure legend. The *P* values <0.05 were considered significant. The reported *n*-values indicate the total number of experiments that were obtained from the number of mice indicated in Supplementary Table 1. For every data set, at least three mice were used per group, unless otherwise indicated. No statistical methods were used to pre-determine the sample sizes but our sample sizes are similar to those reported in previous publications^54^,^59^,^60^.

## Additional information

**How to cite this article:** Parsons, M. P. *et al*. Real-time imaging of glutamate clearance reveals normal striatal uptake in huntington disease mouse models. *Nat. Commun.* 7:11251 doi: 10.1038/ncomms11251 (2016).

## Supplementary Material

Supplementary Information Supplementary Figures 1-13 and Supplementary Table 1.

## Figures and Tables

**Table 1:** GLT-1 expression/function in HD.

Reference	Model (age)	mRNA	Protein	Exogenous glutamate uptake capacity
Lievens *et al*.^15^	R6/2 (8–12 wks)	**↓**	**↓**	**↓** 3H-glutamate uptake
Behrens *et al*.^16^	R6/2 (6–12 wks)	**↓**	**↓**	**↓** 3H-aspartate uptake
Shin *et al*.^19^	R6/2 (10–12 wks)	NA	**↓**	**↓** 3H-glutamate uptake
Miller *et al*.^18^	R6/2 (7 wks)	NA	—	↓ No-net-flux microdialysis slope
Faideau *et al*.^20^	Htt-171–82Q (4–12 wks; astrocyte-specific)	**↓**	**↓**	**↓** 3H-aspartate uptake
Huang *et al*.^17^	YAC128 (3–12 mo)	NA	—	**↓** 3H-glutamate uptake
Present study	YAC128 (1 mo)	NA	NA	↓ 3H-glutamate uptake

Wk, week; mo, month; NA, not applicable; Summary of the literature and new data supporting a role for impaired uptake of exogenous glutamate (or aspartate) in HD pathology. See text for details on the glutamate uptake assay results from the present study. See ref. 53 for a recent review on the different mouse models of HD. —, no change.

**Table 2:** Membrane properties of astrocytes recorded from WT (*B6CBAF1/J*, *n*=9) and *R6/2* (*n*=8) mice.

Genotype	*n*	Cm (pF)	Rm (MΩ)	Tm (ms)	RMP (mV)
WT	9	242.8±47.7	6.9±1.6	903.1±48.9	−88.7±1.4
R6/2	8	86.9±15.8	13.3±2.1	798.3±68.4	−87.6±1.8
*T*-test (*P* value)		0.015*	0.0274*	0.2206	0.6389

Cm, membrane capacitance; Rm, membrane resistance; τm, membrane tau; RMP, resting membrane potential.

All the data are expressed±s.e.m. Unpaired *t*-test. *P<0.05.
