# Supplementary material for: Real-time imaging of glutamate clearance reveals normal striatal uptake in Huntington disease mouse models
Source: Nat Commun. 2016 Apr 7;7:11251. doi: 10.1038/ncomms11251 (PMC4829692; doi:10.1038/ncomms11251)
Supplement: Supplementary Information — Supplementary Figures 1-13 and Supplementary Table 1. [file ncomms11251-s1.pdf]

Supplementary Table 1

Animal ages					
Genotype	*disease stage*	*promoter*	*Inj. age (days)*	*Exp. age (days)*	*n*
FVB/N	N/A	synapsin	29.6 ± 0.7	60.5 ± 2.0	24
YAC128	prior to motor phenotype	synapsin	30.6 ± 0.3	58.9 ± 1.3	14
FVB/N	N/A	GFAP	37.0 ± 0.0	65.0 ± 1.2	3
YAC128	prior to motor phenotype	GFAP	37.0 ± 0.0	65.0 ± 1.2	3
FVB/N	N/A	synapsin	450.0 ± 0.0	477.8 ± 1.7	4
YAC128	clear motor phenotype	synapsin	452.0 ± 0.0	473.3 ± 1.9	3
B6CBAF1/J	N/A	synapsin	33.2 ± 0.4	69.0 ± 2.9	5
R6/2	clear motor phenotype	synapsin	33.2 ± 0.5	68.0 ± 2.4	4

Supplementary Table 1 Average age (±s.e.m.) of all animals used in the present study. Inj. injection; Exp. experiment.

Supplementary Figure 1

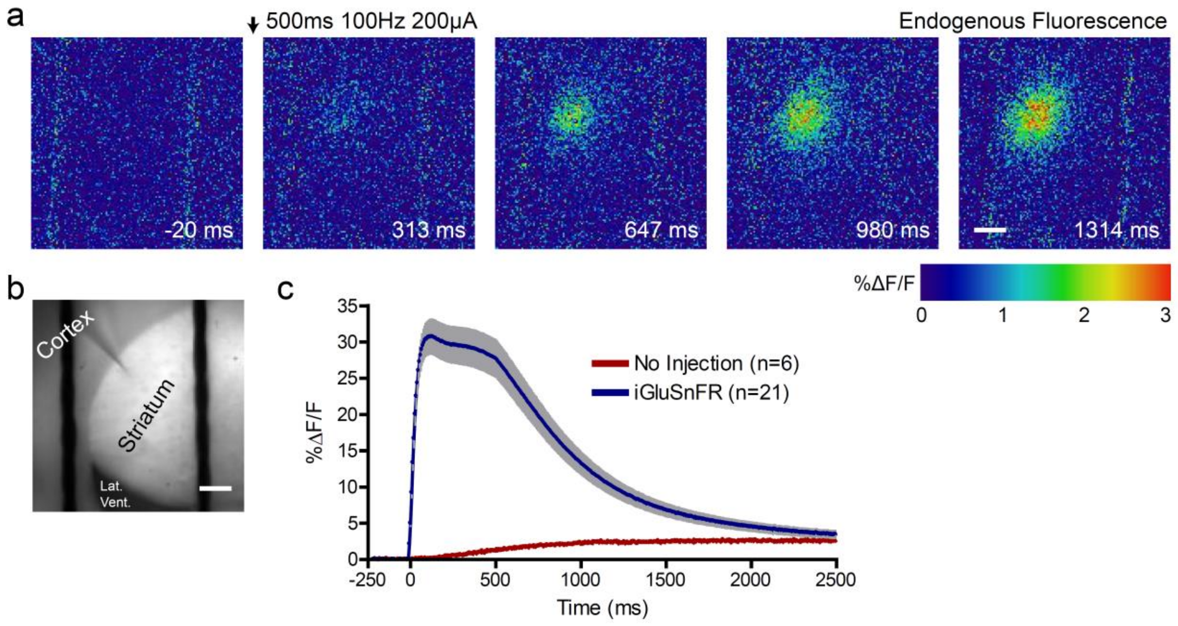

Supplementary Figure 1 Minimal interference of endogenous fluorescence following electrical stimulation. (**a**) Representative frames depicting the fluorescent response (room temperature) in the brain slice from a non-injected *FVB/N* mouse to high frequency stimulation consisting of 50 pulses at 100 Hz. The location of the stimulating electrode is shown in (**b**). (**c**) The endogenous fluorescence response is plotted together with the average response using the same stimulation parameters in brain slices from animals injected with synapsin-iGluSnFR. The increase in endogenous fluorescence was negligible in comparison to the large responses seen in slices from iGluSnFR-injected mice. No endogenous fluorescence change was detected in response to single pulses (not shown). Scale bars = 500 μm

Supplementary Figure 2

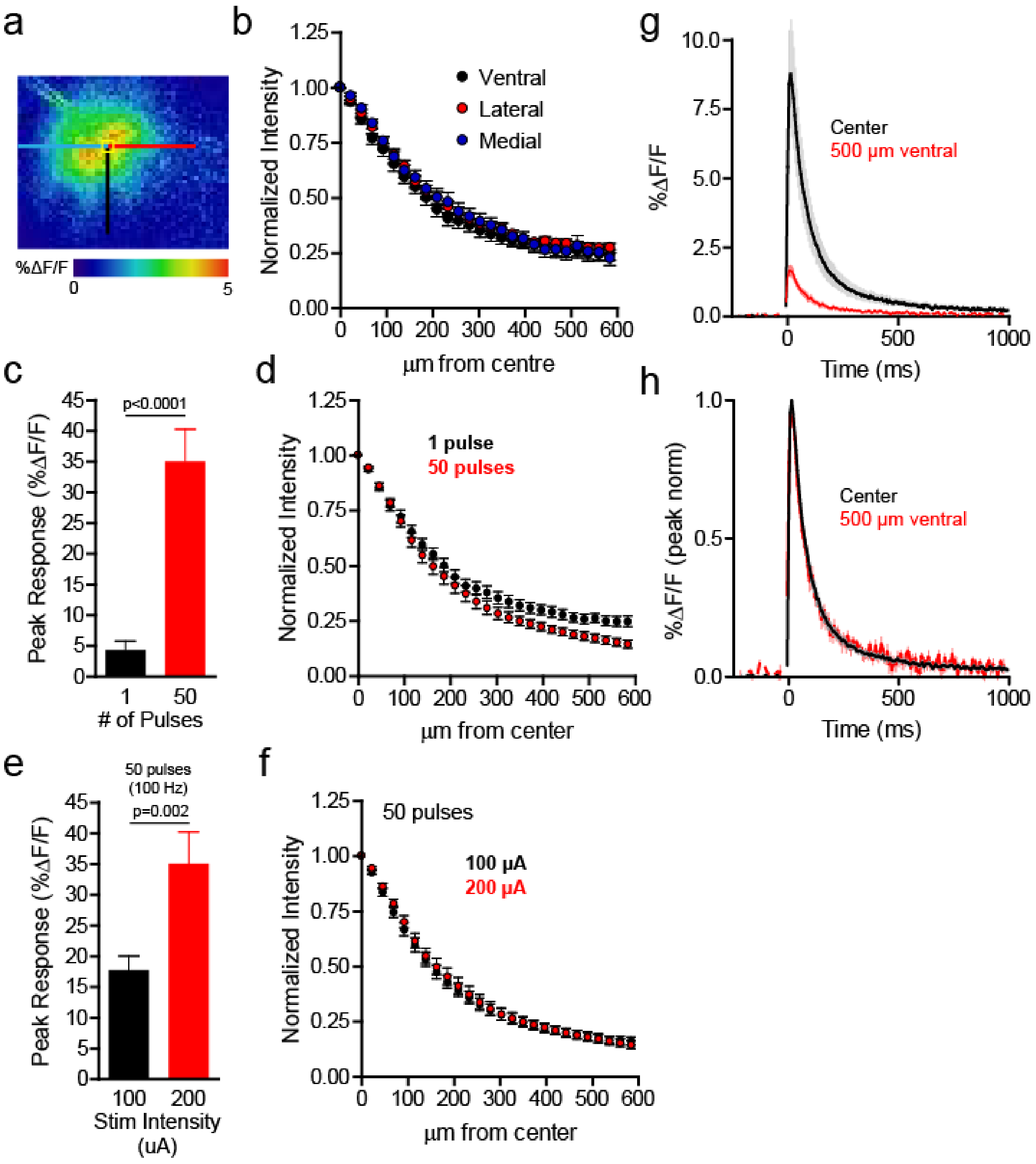

Supplementary Figure 2 Spread of iGluSnFR responses and decay kinetics away from the central region. (**a**) Representative maximum projection image of a typical iGluSnFR response to a single pulse (200 µA) in the dorsal striatum. Lines indicate the sampling regions used to measure spread in the ventral (black), lateral (red) and medial (blue) directions. (**b**) Grouped data showing that the decreasing intensity of the iGluSnFR response with increasing distances from the central point of releases is not significantly different in the ventral, lateral and medial directions (n=23, p=0.460, RM two-way ANOVA); thus, iGluSnFR spread was calculated in the ventral direction for subsequent experiments. (**c**) Bar graph showing that peak responses are increased by increasing the number of pulses (200 µA) from 1 (black) to 50 (100 Hz; red; 1 pulse n=15, 50 pulses n=7, p<0.0001, t-test). (**d**) Despite the increase in peak, there is no increase in the proportional spread of iGluSnFR from the centre following 50 pulses (n values as in **c**, p=0.144, RM two-way ANOVA). (**e**) Bar graph showing that peak responses are increased by increasing the intensity of 50 pulses (100 Hz) from 100 to 200 µA (100 µA n=16, 200 µA n=7, p=0.002, t-test). (**f**) Despite the increase in peak, there is no increase in the proportional spread of iGluSnFR from the centre (n values as in **e**, p=0.797, RM two-way ANOVA). (**g-h**) Grouped data showing the average response of iGluSnFR to a single pulse (200 µA) as measured in a 4x4 region of interest placed either directly at the central point of release (i.e. maximal iGluSnFR response; black) or 500 µm ventral to this central release site (red). The peak-normalized responses can be seen in (**h**). Experiments were performed in *B6CBAF1/J* mice at 32 °C.

Supplementary Figure 3

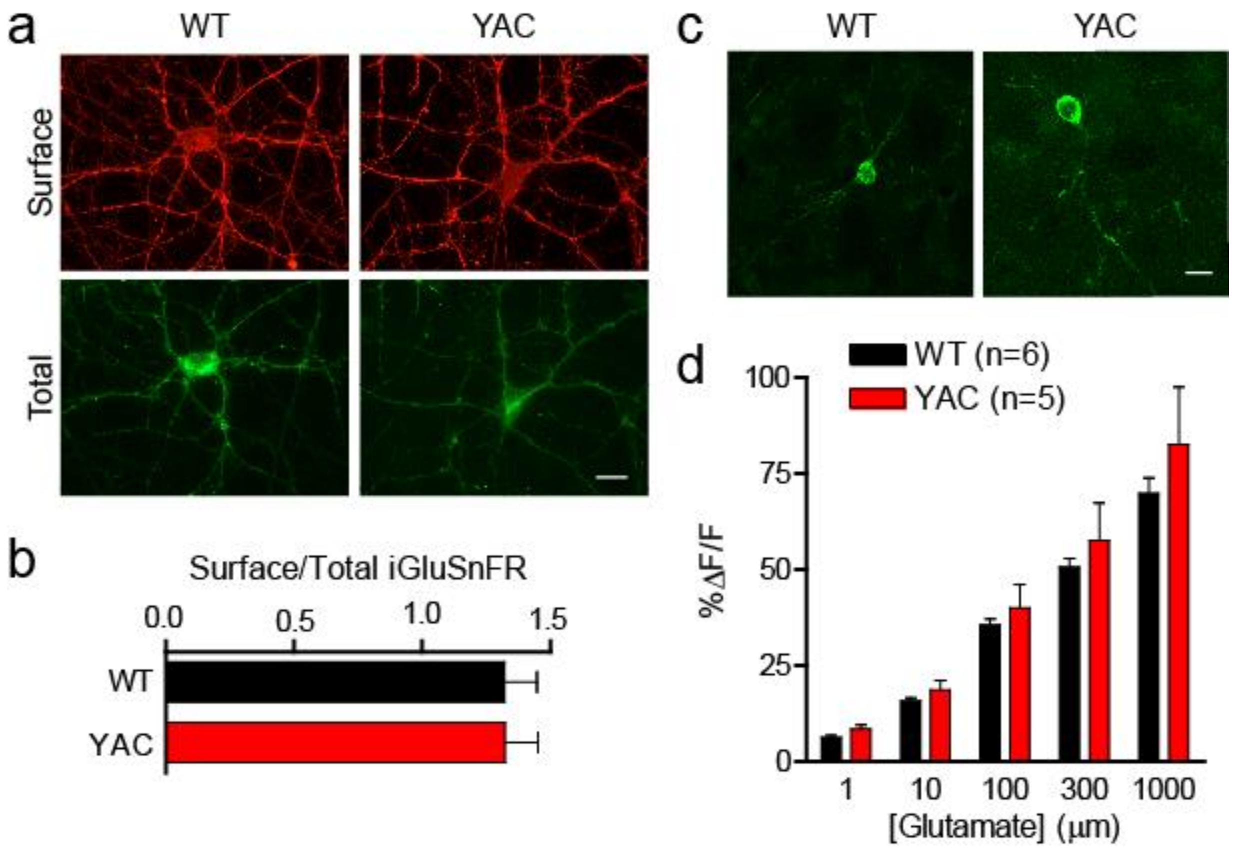

Supplementary Figure 3 iGluSnFR expression and response to exogenous glutamate is not affected by the presence of mutant huntingtin. (**a**) Representative images of striatal neurons co-cultured with cortical neurons. Surface iGluSnFR was amplified with an anti-GFP antibody and Alexa 568 secondary antibody. Cultures from WT (left) and *YAC128* (right) tissues are shown. (**b**) Grouped data of the amplified surface (Alexa 568) staining intensity normalized to the total iGluSnFR intensity (GFP). There was no significant difference between genotypes (WT n=36; *YAC128* n=38, p=0.986, unpaired t-test), suggesting that mutant huntingtin presence does not alter iGluSnFR trafficking to the surface. (**c**) Representative confocal images of iGluSnFR-expressing neurons from WT and *YAC128* striatal sections. Diffuse staining in the soma and dendrites is evident for both WT and *YAC128* neurons. (**d**) iGluSnFR response to increasing

concentrations of exogenous glutamate. There was no significant difference in the response to glutamate in WT and *YAC128* brain slices (WT n=6; *YAC128* n=5; genotype p=0.380, repeated-measures two-way ANOVA). Scale bars = 15 μm

Supplementary Figure 4

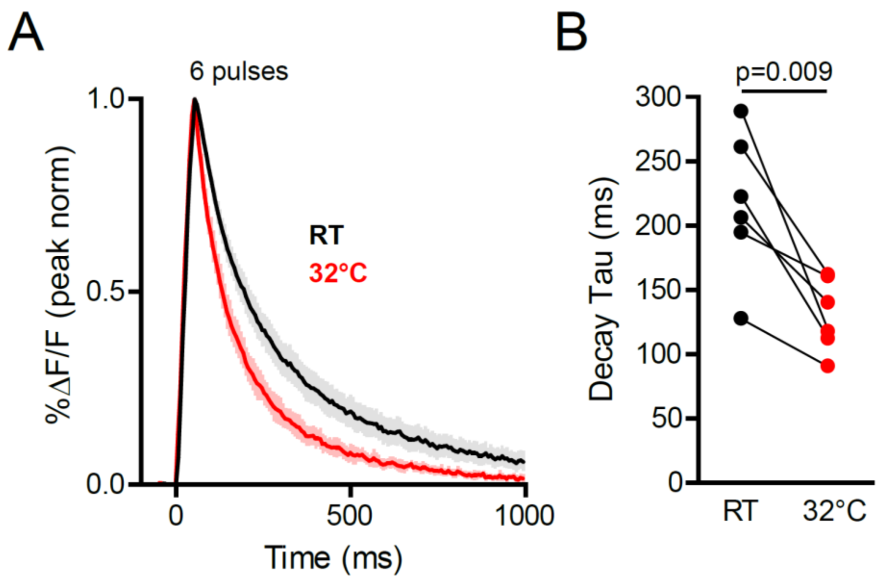

Supplementary Figure 4 iGluSnFR decay tau is temperature-sensitive. (**a**) Grouped data showing the average response to 6 pulses at 100 Hz (100 µA) at room temperature (RT; black) and at 32 ± 1°C (red). Responses, obtained from brain slices of *FVB/N* mice, were recorded first at either RT or 32°C and then switched to the other temperature to measure both responses from the same location. The starting temperature alternated from RT to 32°C between experiments. Responses are normalized (norm) to their peaks for direct comparisons of decay tau. Temperature had no significant effect on response size (RT, 9.8 ± 2.1 %ΔF/F; 32°C, 10.2 ± 3.9 %ΔF/F, n=6, p=0.907). Paired data is shown in (**b**; paired t-test).

Supplementary Figure 5

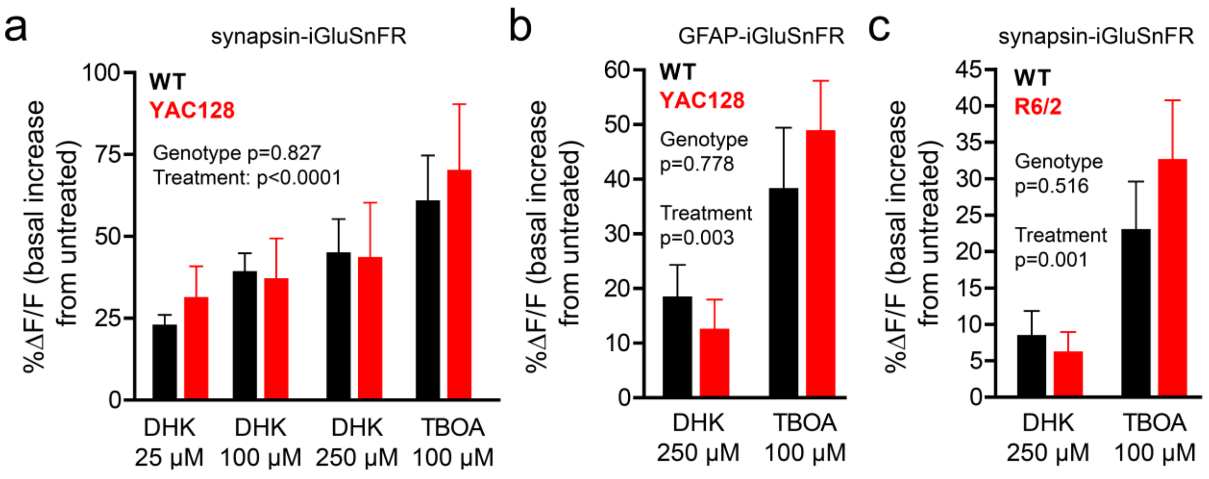

Supplementary Figure 5 Glutamate transporter inhibition increases basal levels of iGluSnFR fluorescence intensity. (**a**) Bar graph showing the average increase in basal, unstimulated synapsin-iGluSnFR intensity in response to 10 minute bath application of the reported concentrations of DHK and TBOA in WT (*FVB/N*, n=5 per bar) and age-matched (2-months) *YAC128* mice (n=5 per bar). (**b**) Same as in (**a**), except iGluSnFR was expressed under the control of the GFAP promoter. WT n=6-7 per bar, *YAC128* n=5-6 per bar. (**c**) Same as in (**a**) except in WT (*B6CBAF1/J*, n=8 per bar) and age-matched (10 weeks) *R6/2* mice (n=6 per bar). Reported statistics were obtained by two-way ANOVA.

Supplementary Figure 6

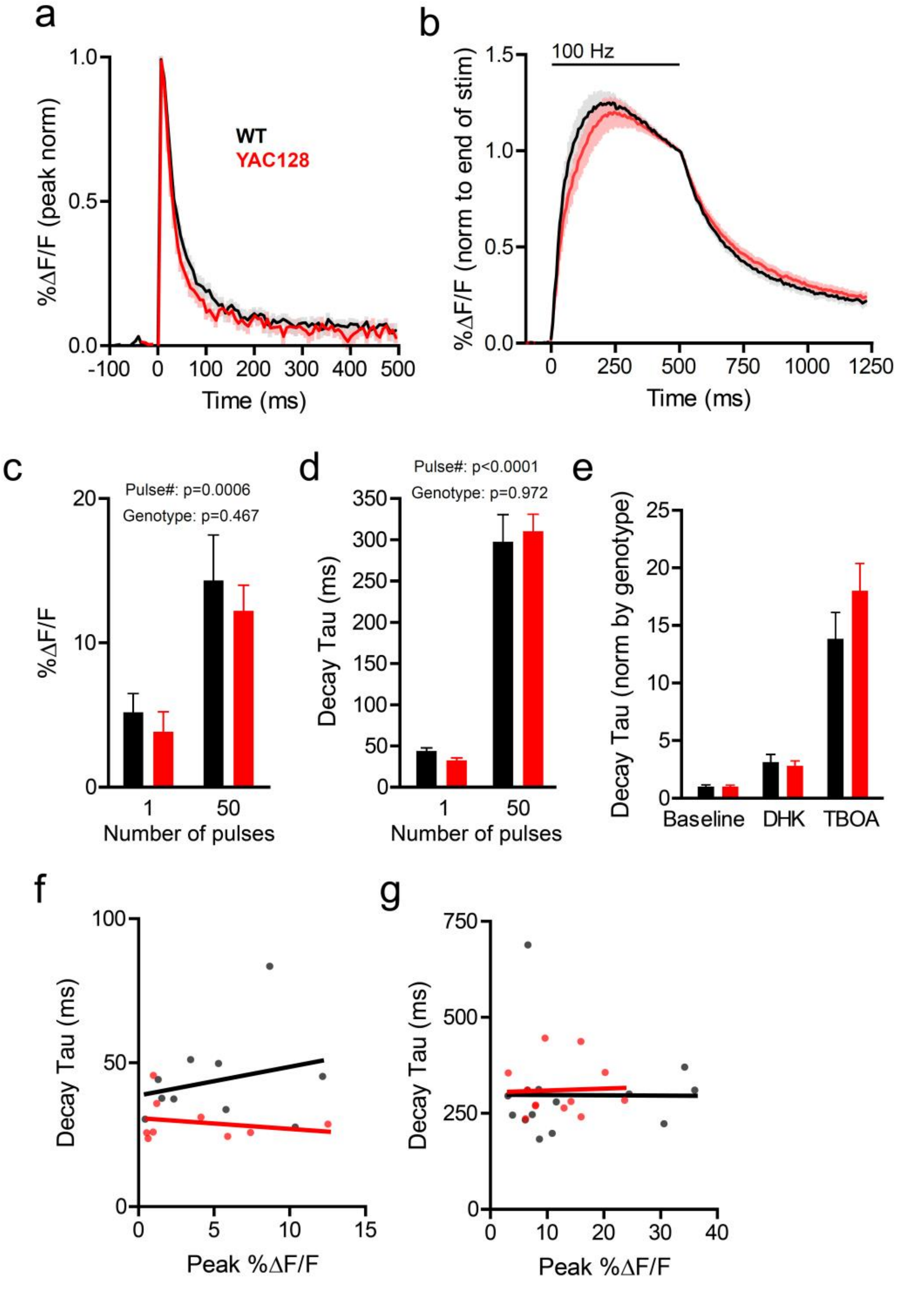

Supplementary Figure 6 Glutamate clearance dynamics in WT and *YAC128* mice at 2 months of age, as measured by iGluSnFR expression in astrocytes. (**a-b**) Grouped data showing the average GFAP-iGluSnFR response (± S.E.M) to a single pulse (**a**; 300 µA) and 50 pulses (**b**; 100 Hz, 200 µA) in the dorsal striatum from WT (*FVB/N*; black) and age-matched, *YAC128* (red) mice. Responses are normalized (norm) to their peaks (**a**) or to their %ΔF/F value at the end of stimulation (**b**). Unlike synapsin-iGluSnFR, in which robust responses were virtually always detected with a single pulse of 200 µA, we found that the single pulse intensity had to be increased to 300 µA to observe the same success rate of obtaining robust GFAP-iGluSnFR responses. (**c-d**) Bar graphs showing the effect of pulse number on the peak (**c**) and decay tau (**d**) of GFAP-iGluSnFR responses (WT n =14-16 per bar; *YAC128* n=12-17 per bar; two-way ANOVA). (**e**) Bar graph showing the fold-increase in GFAP-iGluSnFR decay tau following 10 minute bath application of the GLT-1-specific blocker dihydrokainic acid (DHK; 250 µM) or the non-selective glutamate transporter inhibitor threo-β-Benzyloxyaspartic acid (TBOA; 100 µM). Values are normalized to the genotype average in baseline (untreated) conditions. WT (n=7 per bar; *YAC128* n=5-8 per bar; two-way ANOVA. (**f-g**) Linear regression plots showing no correlation for either WT (black) or *YAC128* (red) between GFAP-iGluSnFR response size and decay tau following a single pulse at 300 µA (**f**; WT r^2=0.066, p=0.475; *R6/2* r^2=0.048, p=0.354) or 50 pulses at 100 µA, 100 Hz (**g**; WT r^2=0.001, p=0.977, *R6/2* r^2=0.002, p=0.891).

Supplementary Figure 7

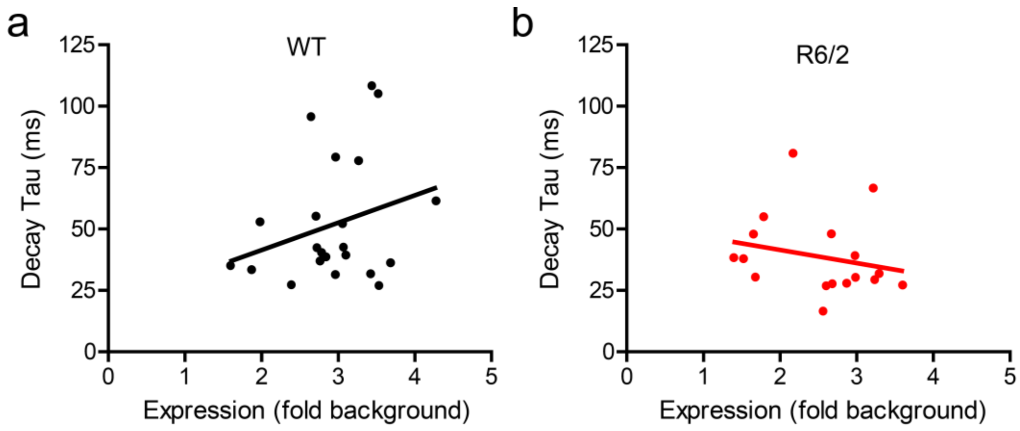

Supplementary Figure 7 iGluSnFR Decay kinetics does not correlate with expression level. (**a-b**) Linear regression plots showing no significant correlation for either WT (**a**; *B6CBAF1/J*; r^2=0.076, p=0.214) or age-matched (10 weeks) *R6/2* mice (**b**; r^2=0.052, p=0.380) between iGluSnFR expression level and decay tau following a single pulse at 200 µA. Expression level was determined by dividing the basal, unstimulated iGluSnFR intensity level at the site of stimulation by the average intensity of three regions of interest that were outside of the iGluSnFR injection region.

Supplementary Figure 8

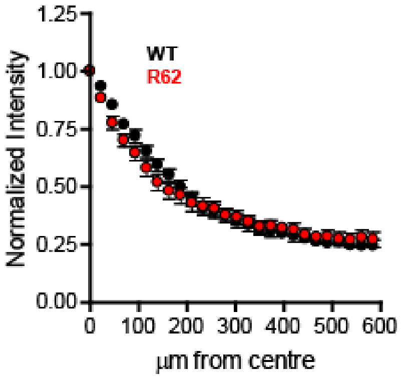

Supplementary Figure 8 The spread of iGluSnFR signals is similar in WT (*B6CBAF1/J*) and *R6/2* striatum at 10 weeks of age. Graph shows the decreasing intensity of iGluSnFR signals with increasing distance from the central region of release. No significant genotype difference was observed in the spread of iGluSnFR signals between WT and *R6/2* mice (WT n=23, *R6/2* n=18, p=0.753, RM two-way AVNOA). Responses were generated by a single pulse (200 µA).

Supplementary Figure 9

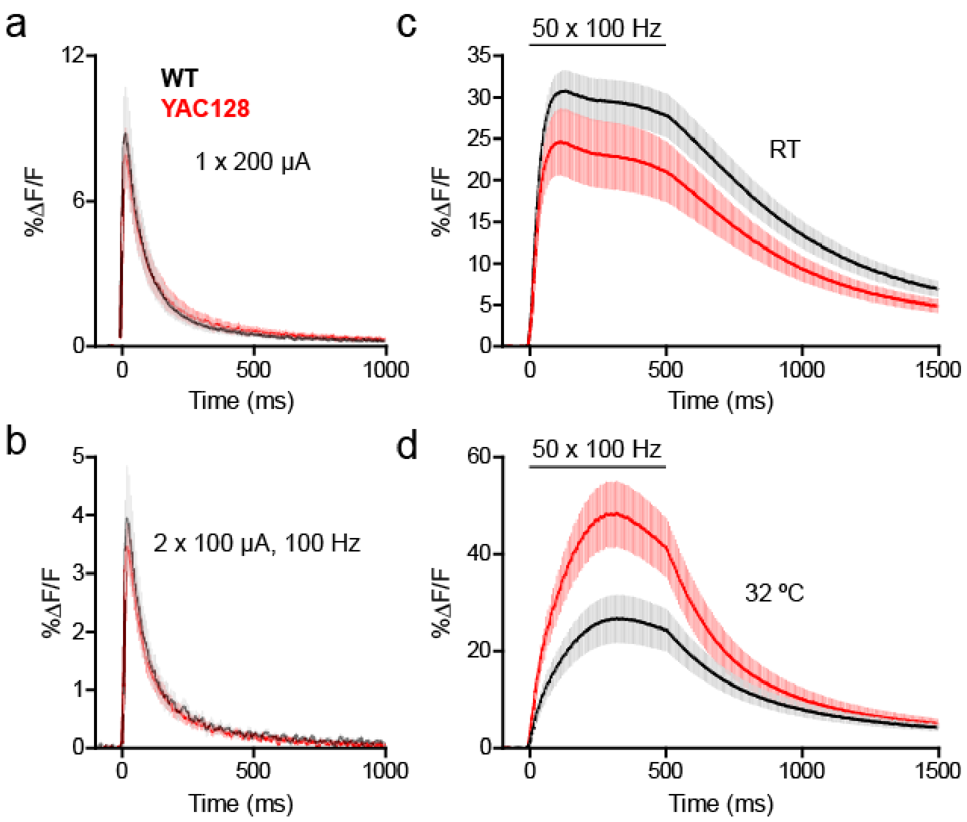

Supplementary Figure 9 Average iGluSnFR responses showing the raw data of the normalized graphs in figure 2. (**a**) Fig 2a (**b**) Fig 2d (**c**) Fig 2g (**d**) Fig 2j.

Supplementary Figure 10

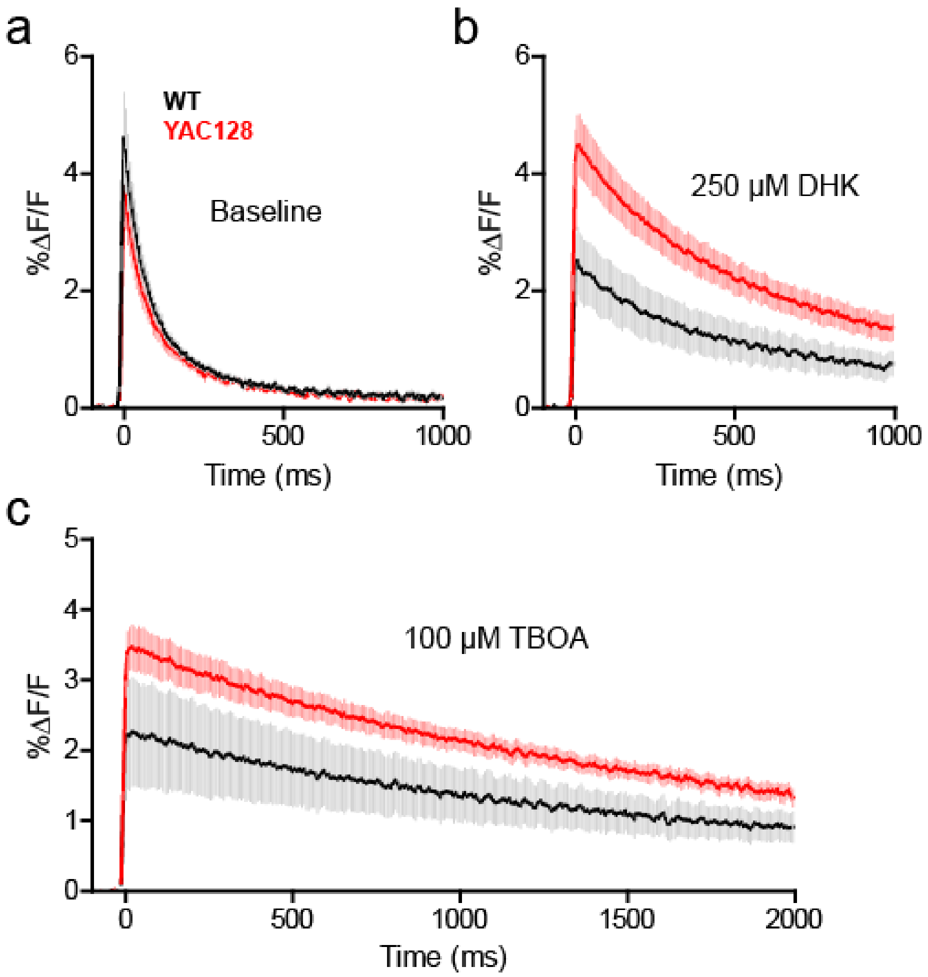

Supplementary Figure 10 Average iGluSnFR responses showing the raw data of the normalized graphs in figure 3. (**a**) Fig 3b (**b**) Fig 3c (**c**) Fig 3d.

Supplementary Figure 11

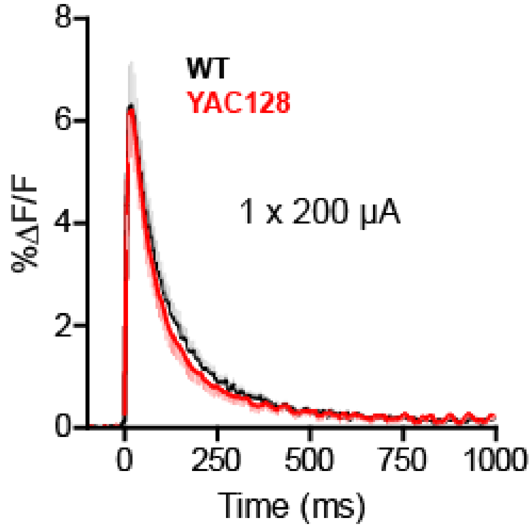

Supplementary Figure 11 Average iGluSnFR responses showing the raw data of the normalized graph in figure 4a.

Supplementary Figure 12

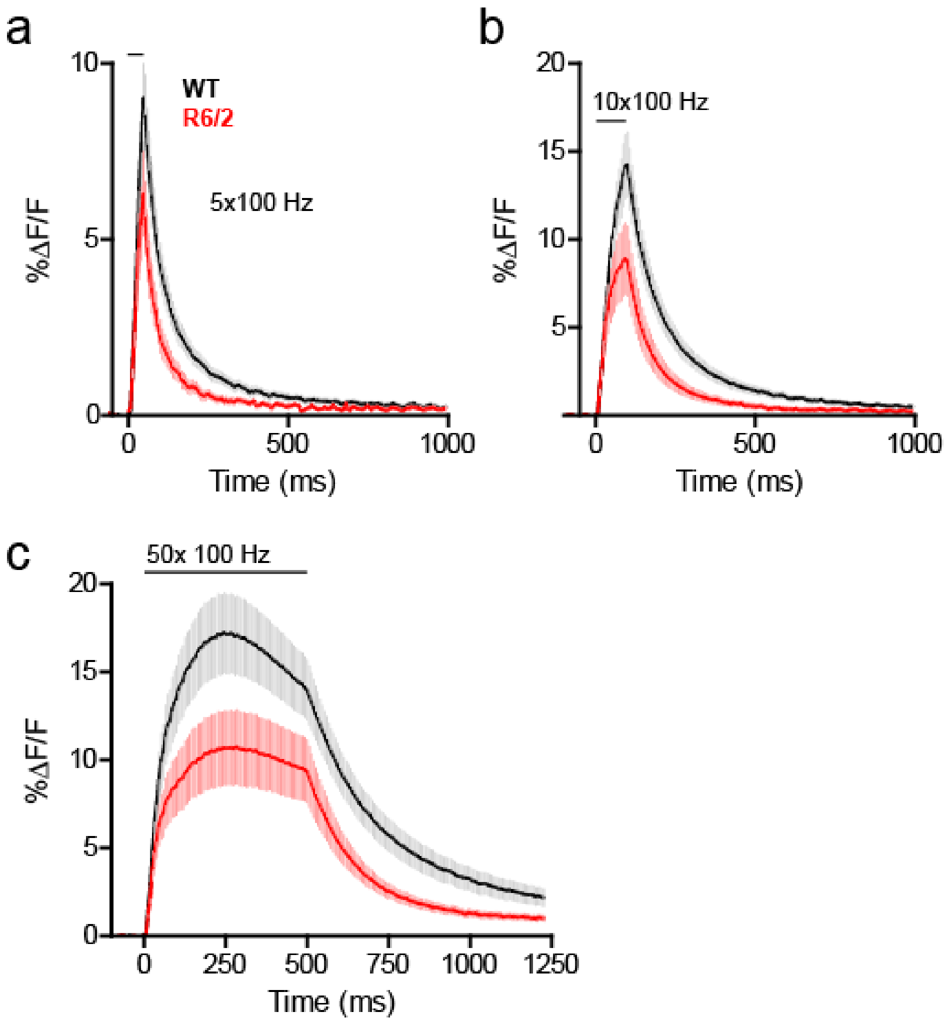

Supplementary Figure 12 Average iGluSnFR responses showing the raw data of the normalized graphs in figure 5. (**a**) Fig 5a (**b**) Fig 5b (**c**) Fig 5c.

Supplementary Figure 13

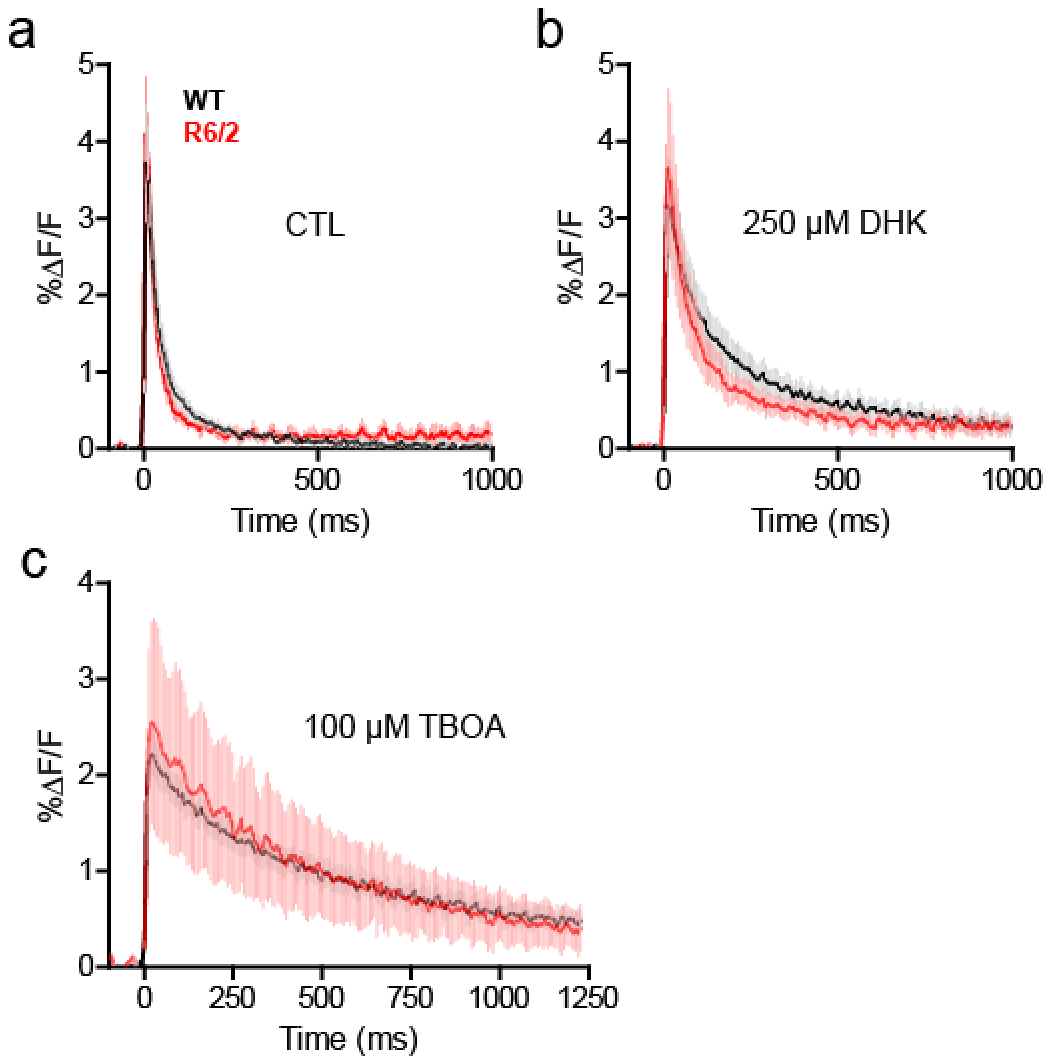

Supplementary Figure 13 Average iGluSnFR responses showing the raw data of the bar graphs in figure 5j.
